# Timeliness of 24 childhood immunisations and evolution of vaccination delay: Analysis of data from 54 low- and middle-income countries

**DOI:** 10.1371/journal.pgph.0003749

**Published:** 2024-11-26

**Authors:** Nieves Derqui, Isobel M. Blake, Elizabeth J. Gray, Laura V. Cooper, Nicholas C. Grassly, Margarita Pons-Salort, Katy A. M. Gaythorpe

**Affiliations:** MRC Centre for Global Infectious Disease Analysis, School of Public Health, Imperial College London, London, United Kingdom; University of Cape Town, SOUTH AFRICA

## Abstract

Vaccination timeliness is often not considered among standard performance indicators of routine vaccination programmes, such as vaccination coverage, yet quantifying vaccination delay could inform policies to promote in-time vaccination and help design vaccination schedules. Here, we analysed vaccination timeliness for 24 routine childhood immunisations for 54 countries. We extracted individual vaccination status and timing from Demographic and Health Surveys data from 54 countries with surveys from 2010 onwards. Individual data was used to estimate age at vaccination for <5 year-old children. Recommended age of vaccination for each country and vaccine was compared to the age at vaccination to determine vaccination delay. The evolution of vaccination delay over time was described using estimates from different birth cohorts. To identify socio-demographic indicators associated with delayed vaccination, we used multivariable Cox regression models with country as random effect and estimated the Hazard Ratio for vaccination with each vaccine-dose for each week post recommended vaccination age. Vaccine coverage at the recommended age was highest for birth and first doses (e.g. 50.5% BCG, 18.5% DTP-D1) and lowest for later doses (e.g. 5.5% DTP-D3, 16.3% MCV-D1, 8.2% MCV-D2). Median delay was lowest for birth doses, e.g. BCG (1 week (IQR: 0 to 4)), and it increased with later doses in vaccination courses: 1 (0, 4) week for DTP-D1 versus 4 (2, 9) weeks for DTP-D3. Although the median delay for each vaccine-dose remained largely constant over time, the range of delay estimates moderately decreased. Children living in rural areas, their countries’ poorer wealth quintiles and whose mothers had no formal education were more likely to received delayed vaccinations. Although we report most children are vaccinated within the recommended age window, we found little reduction on routine immunisation delays over the last decade and that children from deprived socioeconomic backgrounds are more likely to receive delayed vaccinations.

## Introduction

Vaccination schedules are official guidelines on which vaccines are recommended to be administered at which age(s), and as such, are a key component of national immunisation programmes [1]. These schedules are designed to maximise vaccination impact, by facilitating vaccine uptake (for example by recommending simultaneous administration of several vaccines to decrease the number of needed visits to a healthcare facility [2, 3]) and ensuring an optimal immune protection [4]. To boost the immune protection of a vaccine against a Vaccine-Preventable Disease (VPD), vaccination recommendations consider age-specific immune system characteristics, prevalence of the VPD in different age groups, and age-specific disease mortality and morbidity, among other factors [1, 4]. Thus, vaccines given too early may not elicit an immune response and fail to confer protection, while vaccines given delayed may leave populations unprotected and at risk of outbreaks.

Timeliness of immunisation is often not considered in the evaluation of immunisation programmes’ performance. The performance of immunisation programmes is generally assessed using vaccination coverage estimates, calculated as the proportion of people who have received a particular vaccine by a certain age (e.g. at 12–23 months) [5, 6]. This measure usually uses restrictive age reference groups and does not capture whether vaccinations are received at the recommended age or later. However, assessing vaccination timeliness could provide further insight into vaccination performance [7, 8], and inform health policies such as the promotion of timely vaccination (e.g. when and where are vaccination reminders due) or the introduction of new immunisations into the vaccination schedule.

Comprehensive analyses of vaccination timeliness across multiple countries have already been performed [9, 10] using Demographic and Health Surveys (DHS) and Multiple Indicator Cluster Surveys (MICS) data. However, these analyses are relatively old (they were published in 2009 and 2011, respectively), and only included timeliness of vaccination against tuberculosis (Bacillus Calmette-Guérin, BCG), Diphtheria-Tetanus-Pertussis (DTP), polio or with a Measles-Containing Vaccine (MCV). Although more recent studies describing vaccination timeliness are available, they focus on a single country (e.g. China [11], Ethiopia [12, 13], The Gambia [14], India [15, 16] or Kenya [17]), or have a limited vaccine scope [18]. Furthermore, most assessments categorise vaccination timing using “early vaccination”, “timely vaccination” / “not delayed” and “delayed vaccination” grouping [8, 11–13, 19, 20], which does not analyse delay as a continuous variable and thus does not quantify how large or small delays are. Few studies analyse vaccination timing as a continuous variable [9, 10, 14, 21], which facilitates comparison between different research studies, while using “delayed” / “not delayed” categorisation could be biased by different definitions across analyses.

Here we conduct an analysis of vaccination timeliness for 24 childhood immunisations, including the most recently introduced vaccines (such as Hepatitis B, Haemophilus influenzae type b, Pneumococcus and Rotavirus), using recent DHS data published from 2010 up to October 2023 from 54 low- and middle-income countries. We also quantify how vaccination delay has evolved in the last decade across birth cohorts of children born between 2008 and 2021, and identify factors associated with delayed vaccination using survival analysis.

## Methods

### Data sources

#### Demographic and Health Surveys

Individual data on vaccination status and timing of vaccination was extracted from the Demographic and Health Surveys (DHS) programme, accessed 20^th^ October 2023 [22]. Detailed information on the DHS programme procedures has already been published [23]. Briefly, the DHS programme conducts household-level surveys nationally across more than 90 Low- and Middle-Income Countries (LMICs) to monitor health and population indicators. Although surveys are conducted at a household level, health data on each child younger than five years old under the survey’s respondent’s care is recorded individually.

Current DHS standard questionnaires (for DHS phases seven and eight [24, 25]) contain vaccination information for Bacillus Calmette-Guérin (BCG), Diphtheria-Tetanus-Pertussis (DTP), Measles-Containing Vaccine (MCV), Oral Polio Vaccine (OPV) and OPV-Birth Dose (BD), Inactivated Polio Vaccine (IPV), Hepatitis B (HepB) and HepB-BD, *Haemophilus influenzae type b* (Hib), Pneumococcus Vaccine (PCV) and Rotavirus Vaccine (RV) [24, 25]. All information from vaccines included in the standard questionnaire was extracted, including vaccination status and timing of vaccination where available. Children were considered vaccinated if the original DHS-recorded vaccination status was 1 (child was vaccinated and a date of vaccination was seen on a health card), 2 (survey’s respondent recalled the child was vaccinated but no health card was seen) or 3 (child was vaccinated according to their health card but no date was recorded), and unvaccinated if the code was 0 (child was unvaccinated). When available, timing of vaccination was extracted with the exact date of vaccination (day, month and year).

Alongside individual children’s data, other household-level demographic and socioeconomic indicators are recorded in the DHS surveys, such as place of residence (rural versus urban), occupation and level of formal education of the survey’s respondent, or wealth quintile with respect to that country. Data on demographic and socioeconomic indicators previously identified to be associated with vaccination delay [13, 15, 26] was extracted and recoded as indicated on S1 Table.

All countries participating in the DHS programme that had one or more surveys conducted between 2010 and 2023 were considered in the study but only the last survey-data of each country was downloaded. Countries with no vaccination information (Colombia) were excluded, as well as those with less than 2,000 surveyed children (Albania and Armenia) since, consequently, they would then have even smaller numbers of children with vaccination and vaccination age data. The complete list of countries and surveys included in the analyses can be found on S2 Table.

#### WHO Immunisation Data portal

Data on the recommended age of vaccination (hereby “recommended age” for short) for each country and vaccine dose was obtained from the World Health Organisation (WHO) Immunisation Data portal, accessed 27^th^ October 2023 [27] (S1 Fig). Note only the latest vaccination schedule for each country (currently in use) was available, which may or may not reflect the recommended age of vaccination on the year the DHS surveys were conducted on each country. Where possible, recommended age was extracted in weeks. If the recommended age for vaccination was available in months, we assumed a month has 4.3 weeks.

Data on the year of introduction of each vaccine and dose in each country was also extracted [27] (S2 Table). The year of vaccination introduction was used to determine if each vaccine had been introduced in each country by the time the DHS survey was conducted. All countries were considered to have introduced, by the time of the DHS survey, vaccination with BCG, all three doses of DTP and OPV, and the first dose of MCV, and thus all children could have vaccination data on these immunisations. For HepB, Hib, IPV, MCV-D2, PCV and RV, we assumed children would only have vaccination data if that vaccination was introduced at least one year before the DHS survey.

### Definitions

#### Age at vaccination

DHS surveys, since inception and until phase six, report birth month and year. From DHS phase seven (introduced in 2013) onwards, the exact day of birth has been reported [24, 25]. In our included data, 20% of children completed a phase six questionnaire, 71% completed a phase seven, and 9% completed a phase eight questionnaire. Thus, the exact day of birth was available for 80% of children, and for the remaining 20%, the 15^th^ of each of month was used as the default day of birth. “Age at vaccination” (in weeks, rounding to the nearest integer number) was calculated using the children’s date of birth and the exact date of vaccination for each vaccine. Because of the assumption around the day of birth for 20% of data, the lowest boundary for age at vaccination allowed was minus two weeks. If the date of vaccination was more than two weeks earlier than the date of birth, the data was omitted.

#### Vaccination delay

We defined vaccination delay for each vaccine and dose as the difference between the “age at vaccination” and the “recommended age”, matching each child to their country’s recommended age for each vaccine and dose [27]. Note that, although, we term “delay” the numerical difference between the “age at vaccination” and the “recommended age”, this estimate may be negative indicating that a child was vaccinated before the recommendation.

### Analyses

Analyses were restricted to live children with available vaccination age data on any childhood immunisation. To allow for the measurement of large delays of up to one year, we right-censored children whose time between each recommended vaccination date (calculated as the date of birth plus the recommended vaccination age in that country) and survey date was smaller than a year, hereby termed “buffer time”. A two-year buffer time threshold for censoring was also considered in a sensitivity analysis, which produced similar vaccination delay estimates (available online at https://github.com/NDerqui/Delay_Vaccination_Paper).

Using the “age at vaccination” data, we estimated the cumulative proportion of children vaccinated at each week of age for each vaccine and dose. We termed these coverage-per-week-of-age estimates, which were obtained at a country level and aggregating estimates for all countries. Using the coverage-per-week-of-age estimates, “initial coverage” was defined as the coverage at each vaccine and dose’s recommended vaccination age and “final coverage” as the coverage at five years old (age limit of surveyed children). Summary estimates of vaccination delay were obtained for each vaccine and dose, aggregating all countries’ and also per country. To explore the evolution over time, descriptions of vaccination delay per birth cohort were performed. Each birth cohort was defined as children born in each calendar year.

Previous studies reported that some risk factors associated with delayed vaccinations [7, 8], and thus we analysed whether demographic and socioeconomic indicators recorded in the DHS surveys could be associated with increased vaccination delay. A survival analysis was performed to estimate the probability of being vaccinated by each week post recommended age with each vaccine using the cumulative distribution function F(t) = Pr(T≤t) = 1-S_KM_(t), where t represents each week post vaccination recommendation age and S_KM_ the Kaplan-Meier survival function [28]. To estimate vaccination Hazard Ratio (HR) and identify delayed vaccination indicators for each vaccine and dose, we built a multivariable Cox regression model with country as a random effect. Each multivariable Cox model started with a full model that included all demographic and socioeconomic covariates (except possession of a health card and place of residence, due to low data availability in some categories). Backwards and forward stepwise covariates’ selection was performed, and both methods returned almost identical results (S3 Table), which were also very similar across the different vaccines. “Sex at birth” was the variable most often left out, yet it was selected for seven models. “Wealth quintile”, “mothers’ level of education” and “mothers’ husband’s occupation” were selected in all models. For simplicity and comparability of the different vaccines’ models, we present the results of the full multivariable model (i.e. including all covariates) for all vaccines.

Next, we hypothesised that children receiving first doses (e.g. DTP-D1) delayed would be more likely to lose contact with immunisation services. The association between delay in receiving the first dose and completeness of the vaccination course was explored using logistic regression models. We estimated the Odds Ratio (OR) for receiving second and third doses (using a binary indicator for vaccinated yes or no) as a function of each week of delay in receiving the first dose of that vaccine.

Analyses were conducted using R, version 4.2.3 [29]. The “survival” package [30] was used to perform the survival analysis, and the “coxme” package [31] to conduct a Cox regression with a random effect. The “selectCox” [32] and “stepwiseCox” [33] functions were used to perform backwards and forward variable selection respectively in the multivariable Cox model. All plots were generated using “ggplot2” [34] and “ggridges” [35]. Further information (code and extended results from sensitivity analysis) is available at: https://github.com/NDerqui/Delay_Vaccination_Paper.

### Data source ethics declaration

DHS data is a public and open source, available for research purposes after registration [22]. To download the data, we sought and received approval from the DHS programme. Institutions conducting and managing DHS surveys sought written informed consent from all participants, as per DHS statements and procedures. Authors did not have access to information that could individually identify participants. WHO data is open to download from their website [27].

## Results

### Description of data

In total, data for 743,694 live children from 54 countries included in 54 DHS surveys were considered in the analyses (S4 Table); 379,193 (51%) were male and 364,501 (49%) were female. Children’s median age at time of survey was 30 months (IQR: 14 to 45). Most countries included between 5,000 and 12,000 children in their surveys. India and Nigeria were the countries with the highest number of children (224,218 (30.1% of all children) and 41,358 (5.6%), respectively) (S2 Fig). Among all surveys, there were children born between 2006 and 2022 (S4 Table). However, less than 10,000 children born in 2006, 2007 and 2022 were included in the surveys; therefore, estimates of vaccination delay evolution were omitted for these three birth cohorts.

We assumed all countries had introduced vaccination with BCG, all three doses of DTP and OPV, and the first dose of MCV (S5 Table). Additionally, by the time of their respective survey, all 54 (100%) countries had introduced vaccination with doses 1 to 3 of Hepatitis B, 20 (37%) with Hepatitis B Birth Dose, 53 (98%) with all three doses of Hib, 30 (56%) with IPV-D1, 4 (7%) with IPV-D2, 34 (63%) with MCV-D2, 34 (63%) with all three doses of PCV and 30 (56%) with all three doses of RV. No country had introduced all immunisations analysed in the study. Among children from countries in which each vaccine had been introduced, the percentage of these children who then had vaccination data with each vaccine ranged from 2.1% (IPV-D2) and 24.31% (IPV-D1) to 68.25% (BCG) (S5 Table). For all vaccinations, buffer time between recommended date of vaccination and survey was ≥1 year for at least 60% of the children with vaccination data (S5 Table). Among children with ≥1 year buffer time, no children vaccinated with IPV-D2 were captured in the surveys. Among all vaccinated children, the percentage who then had vaccination date data, and thus were further included in the age at vaccination and vaccination delay analyses, ranged from 57.58% (for IPV-D1) and 81.04% (for RV-D3) (S5 Table).

To assess the representativeness of the children included in the analyses, we compared those included (i.e. children with information on vaccination date) to those excluded (i.e. children without information on vaccination date). The only noted demographic or socioeconomic difference between those two groups (measured as a >5% difference in cross-tabulations for demographic and socio-economic categories) was the possession of a health card (S3 Fig).

### Vaccination coverage per week of age

**Fig 1:**
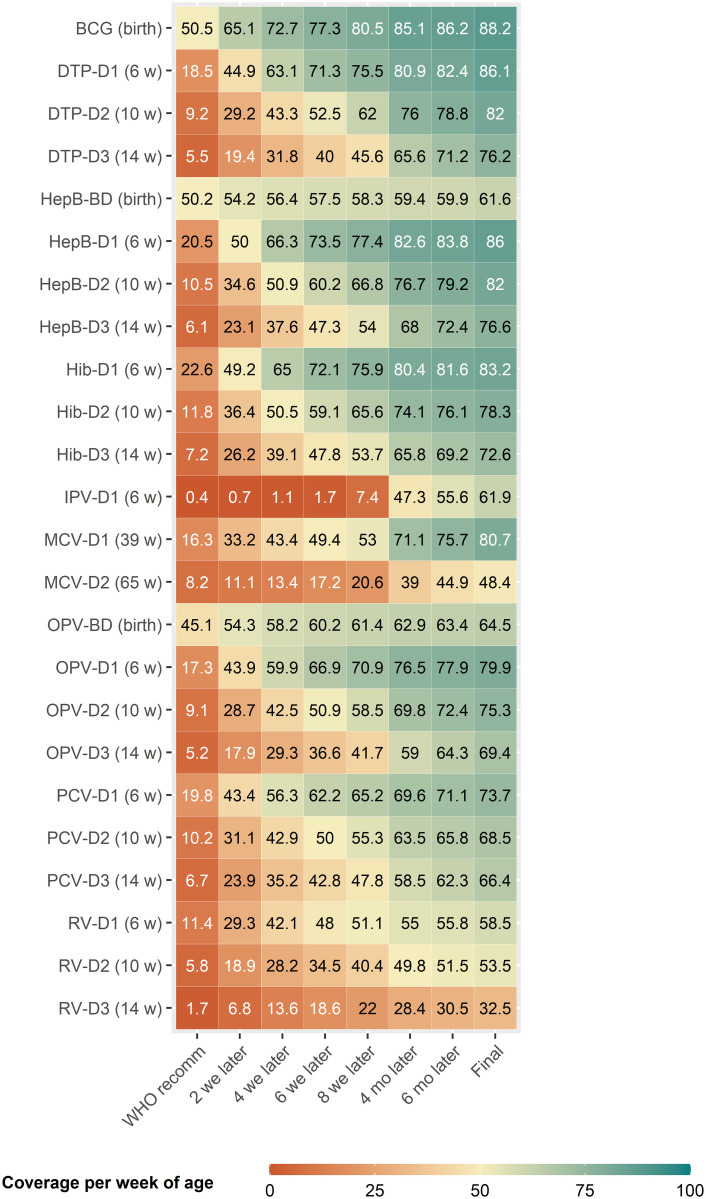
Vaccination coverage per week of age. Coverage per week of age is calculated as the cumulative proportion of children vaccinated at each week of age. General coverage estimates including data for all countries are shown for the WHO recommended vaccination week of age for each vaccine and dose (WHO recomm); two, four, six, and eight weeks after; four and six months after; and at five years of age (final coverage). WHO recommended vaccination age for each vaccine and dose is noted in parenthesis next to each immunisation [26]. Abbreviations: BCG, Bacillus Calmette-Guérin; BD, Birth Dose; D1/2/3, Doses 1, 2 or 3; DTP, Diphtheria-Tetanus-Pertussis; HepB, Hepatitis B vaccine; Hib, Haemophilus influenzae vaccine; IPV, Inactivated Polio Vaccine; MCV, Measles-Containing Vaccine; OPV, Oral Polio Vaccine; PCV, Pneumococcal Vaccine; RV, Rotavirus vaccine; WHO, World Health Organisation.

Initial coverage, defined as the coverage at each vaccine’s recommended age, was highest for birth doses (e.g. 50.5% for BCG and 50.2% for Hep-BD) (Fig 1), and coverage at their respective recommended ages decreased with the later the vaccine was in the schedule; for example, 18.5% for DTP-D1, 9.2% for DTP-D2 and 5.5.% for DTP-D3 (Fig 1). Initial coverage was lowest for RV (11.4% for RV-1, 5.8% for RV-2 and 1.7% for RV-3) (Fig 1).

Final vaccination coverage reached, defined as coverage at the age of five, ranged from 32.5% for RV-D3 to 88.2% for BCG (S5 Table, Fig 1). First doses in vaccination courses achieved a higher final coverage than later doses; for example, DTP-D1 coverage was 86.1% versus 76.2% for DTP-D3, and 80.7% for MCV-D1 versus 48.4% for MCV-D2. However, despite the higher initial coverage, HepB-Birth Dose and OPV-Birth Dose had lower final coverages than the corresponding first doses of their courses (S5 Table, Fig 1).

Additionally, birth and first doses in vaccination courses achieved a ≥50% coverage between two and four weeks after the recommended age (Fig 1). For example, BCG coverage at two weeks after recommendation was 65.1% and DTP-D1 coverage at four weeks after recommendation was 63.1%. In contrast, later doses in vaccination schedules only achieved a ≥50% coverage after four weeks post-recommendation: DTP-D3 coverage at four weeks after recommendation was 31.8% and 65.6% at four months after recommendation (Fig 1). For DTP-D3, IPV-D1, MCV-D1, and OPV-D3, 5% of children were vaccinated more than six months after the recommended age: e.g. final coverage for DTP-D3 was 76.2% but only 71.2% at six months after the recommended vaccination age (Fig 1).

Coverage per week of age per country can be found in the supplementary materials (S4–S6 Figs). Although we observed the general trend that later doses had lower initial and final coverages across countries, specific country estimates varied substantially. Burundi, Egypt and Rwanda present faster vaccination uptakes than other countries, as most of the children are vaccinated within a few weeks of recommendation regardless of the immunisation. In contrast, in Angola, Chad, Guinea or Nigeria vaccination uptake was slower and achieved lower coverages for all vaccines. Across all countries, MCV-D2 and the RV course experience the lowest vaccination uptakes and reach smaller coverages (for example, coverage per week of age estimates for India or Uganda MCV-D2 and RV are very different to those of BCG or DTP).

### Delay in vaccination

**Fig 2:**
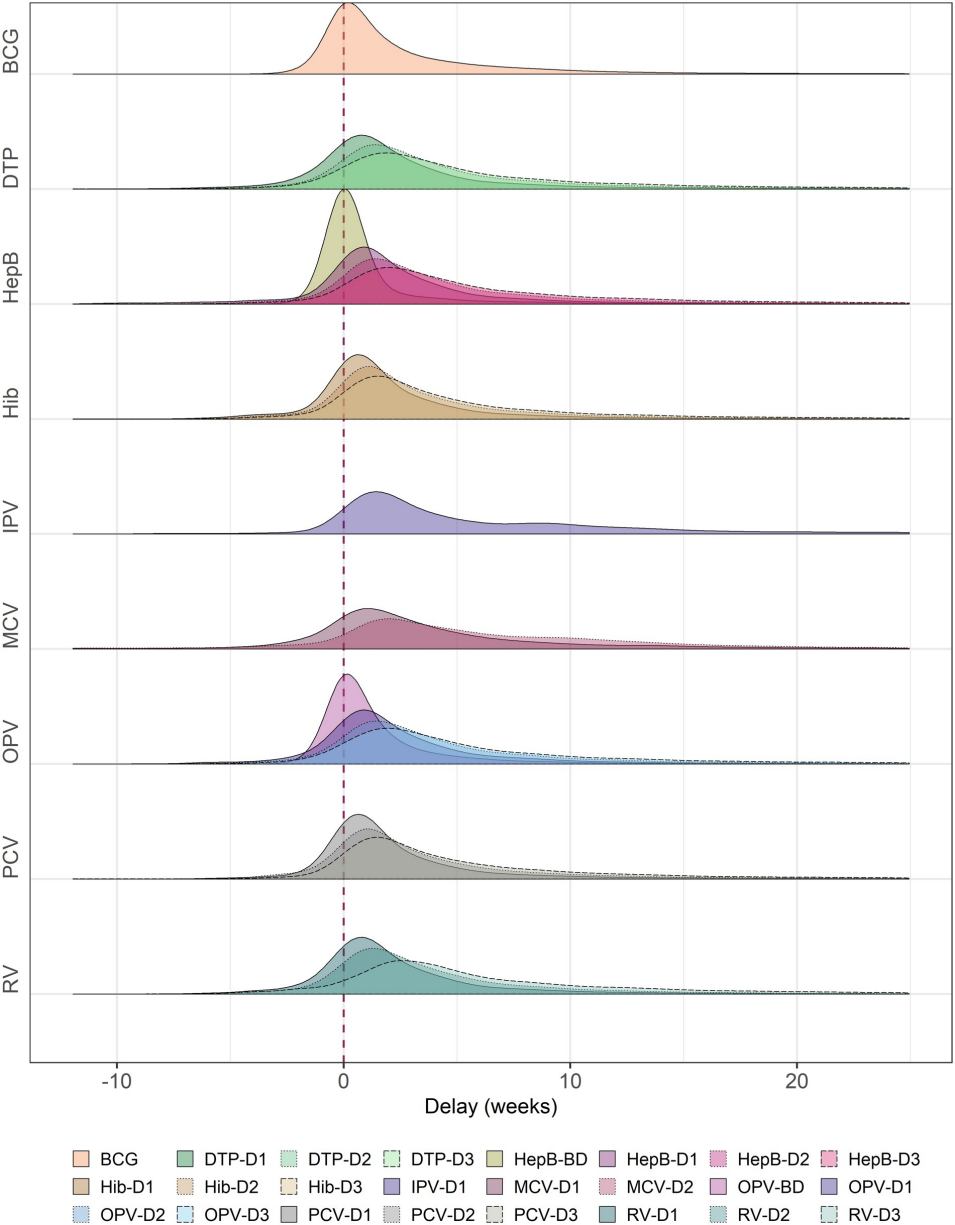
Vaccination delay distribution for each vaccine and dose. The density distribution for vaccination delay was generated by “ggridges” [35]. Vaccination delay (on x-axis) is defined as the difference between age at vaccination and recommended age for vaccination. Dashed line (x = 0) indicates no delay or early vaccination (timely vaccination). All measures of vaccination delay are indicated in weeks. Note x-axis was limited to -12 to 25 weeks. Abbreviations: BCG, Bacillus Calmette-Guérin; BD, Birth Dose; D1/2/3, Doses 1, 2 or 3; DTP, Diphtheria-Tetanus-Pertussis; HepB, Hepatitis B vaccine; Hib, Haemophilus influenzae vaccine; IPV, Inactivated Polio Vaccine; MCV, Measles-Containing Vaccine; OPV, Oral Polio Vaccine; PCV, Pneumococcal Vaccine; RV, Rotavirus vaccine.

Median vaccination delay was lowest for birth doses: BCG (1 week (IQR: 0 to 4)), HepB-BD (0 (0 to 0)) and OPV-BD (0 (0, 2)) (Fig 2, S6 Table). The later the dose in the vaccine course, the larger the delay observed; for example, median delay was 1 (0, 4) weeks for DTP-1 versus 4 (2, 9) weeks for DTP-3, and 1 (0, 4) for OPV-1 versus 4 (2, 9) for OPV-3 (Fig 2, S6 Table). Furthermore, later doses in vaccine courses also presented longer tails in the delay distribution (Fig 2). The same tendency was observed at a country level, with later vaccines such as courses’ third doses and MCV doses being received with an increased delay (S6 Table). However, there was also a wide variety of delays observed across countries: Chad, Mali, Mauritania or Yemen had higher delays across different vaccinations while in Burundi or Rwanda vaccination delay was very small across all immunisations. Across several countries, highest median delays are observed for IPV-D1: 13 (10, 18) weeks in Bangladesh, 13.5 (3, 25) weeks in Guinea and 29.5 (14, 39) in Uganda.

**Fig 3:**
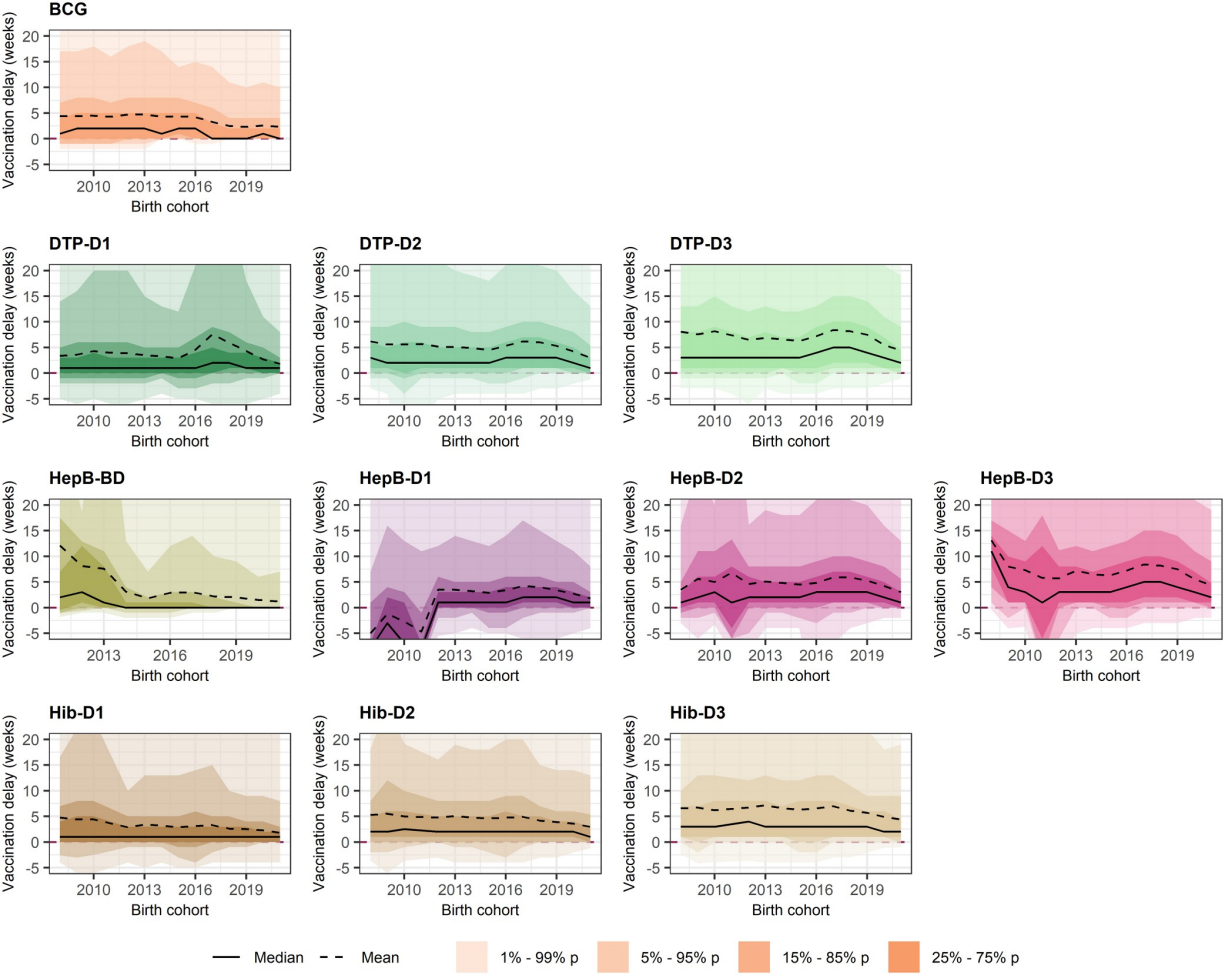
Evolution of mean, median and percentiles of vaccination delay for each vaccine and dose. Vaccination delay is defined as the difference between age at vaccination and recommended age for vaccination. Median and mean vaccination delay are shown as a solid and dashed line, respectively, on y-axis for each birth cohort (on x-axis). The vaccination delay 1^st^-99^th^, 5^th^-95^th^, 15^th^-85^th^ and 25^th^-75^th^ (IQR) percentiles for each birth cohort are shown as ribbons with increasing fill intensity. All measures of vaccination delay are indicated in weeks. Note y-axis was limited to -5 to 20 weeks. Abbreviations: BCG, Bacillus Calmette-Guérin; BD, Birth Dose; D1/2/3, Doses 1, 2 or 3; DTP, Diphtheria-Tetanus-Pertussis; HepB, Hepatitis B vaccine; Hib, Haemophilus influenzae vaccine; IQR, Interquartile Range.

**Fig 4:**
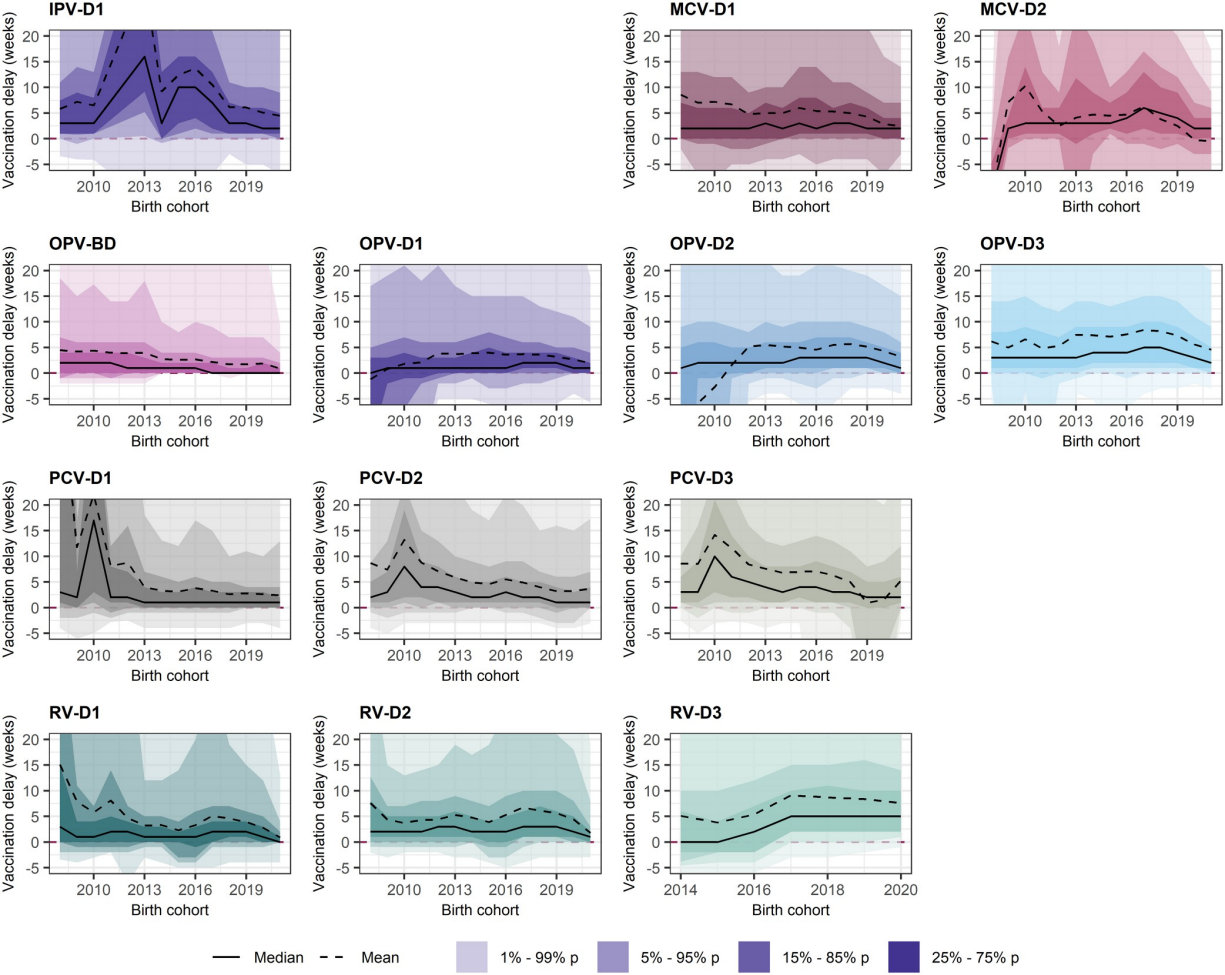
Evolution of mean, median and percentiles of vaccination delay for each vaccine and dose. Vaccination delay is defined as the difference between age at vaccination and recommended age for vaccination. Median and mean vaccination delay are shown as a solid and dashed line, respectively, on y-axis for each birth cohort (on x-axis). The vaccination delay 1^st^-99^th^, 5^th^-95^th^, 15^th^-85^th^ and 25^th^-75^th^ (IQR) percentiles for each birth cohort are shown as ribbons with increasing fill intensity. All measures of vaccination delay are indicated in weeks. Note y-axis was limited to -5 to 20 weeks. Abbreviations: BD, Birth Dose; D1/2/3, Doses 1, 2 or 3; IPV, Inactivated Polio Vaccine; IQR, Interquartile Range; MCV, Measles-Containing Vaccine; OPV, Oral Polio Vaccine; PCV, Pneumococcus Vaccine; RV, Rotavirus vaccine.

A small (<5 weeks) reduction of median vaccination delay was observed among the most recent birth cohorts (Figs 3 and 4). However, despite the median vaccination delay and IQR for all vaccines remained largely the same across birth cohorts, the 5^th^-95^th^ and 15^th^-85^th^ percentile estimates moderately reduced among recent birth cohorts (Figs 3 and 4). This reduction in the maximum delay measured also reduced the average vaccination delay for most vaccines; for example, mean vaccination delay for BCG was 4.4 weeks for children born in 2008 and 2.3 weeks for children born in 2021, and MCV-D1 mean vaccination delay was 8.6 weeks for 2008 children and 2.5 weeks for 2021 children. Nevertheless, this reduction in the mean vaccination delay was moderate for BCG, DTP and OPV. On the other hand, the decrease in mean vaccination delay was more pronounced for more recently introduced vaccines such as HepB, MCV-D2, PCV and RV (Figs 3 and 4): mean vaccination delay for PCV-D1 was 11.8 weeks for 2009 children versus 2.4 weeks for 2021 children, and 7.9 weeks for 2009 children versus 0.9 weeks for 2021 children for RV-D1. A peak of increased vaccination delay was observed for DTP-D1 in 2017.

### Indicators for delayed vaccination

**Fig 5:**
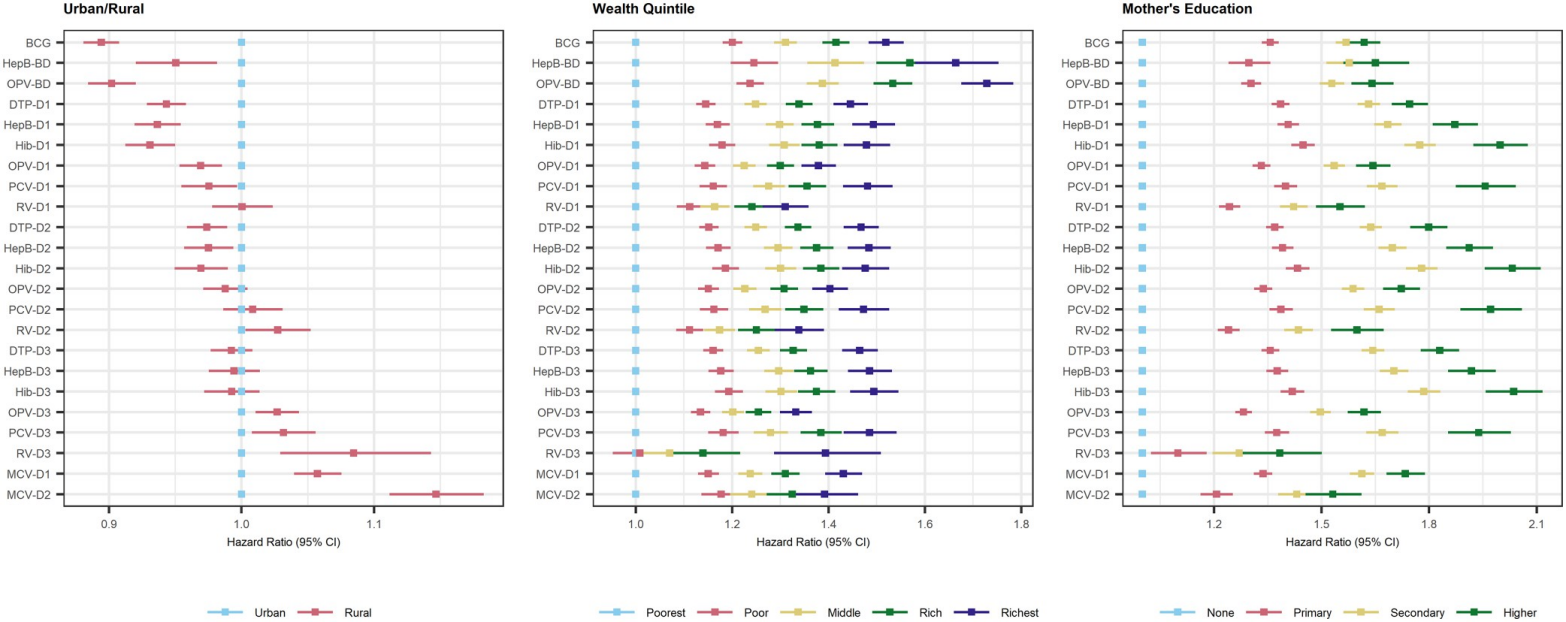
Multivariate analysis of demographic and socioeconomic indicators associated with vaccination delay. Hazard Ratio (and 95% Confidence intervals) for vaccination with each vaccine and dose according to each covariate is represented on the x-axis. Vaccines (on the y-axis) are ordered according to WHO recommended vaccination age. Abbreviations: BCG, Bacillus Calmette-Guérin; BD, Birth Dose; D1/2/3, Doses 1, 2 or 3; DTP, Diphtheria-Tetanus-Pertussis; HepB, Hepatitis B vaccine; Hib, Haemophilus influenzae vaccine; HR, Hazar Ratio; IPV, Inactivated Polio Vaccine; MCV, Measles-Containing Vaccine; OPV, Oral Polio Vaccine; PCV, Pneumococcus Vaccine; RV, Rotavirus vaccine; WHO, World Health Organisation.

Children living in a rural area had a lower vaccination Hazard Ratio (HR) for birth doses and first doses compared to those living in an urban area (Fig 5): HR for vaccination with BCG was 0.89 (95% CI: 0.88, 0.91), 0.95 (0.92, 0.98) with HepB-BD and 0.94 (0.93, 0.96) with DTP-D1. However, children living in a rural area were also associated with a higher HR for being vaccinated with the later doses in the schedule than children living in a urban area: courses’ third doses (e.g. 1.03 (1.01, 1.04) for OPV-D3 and 1.03 (1.01, 1.06) for PCV-D3) and the MCV course (1.06 (1.04, 1.07) for MCV-D1 and 1.15 (1.11, 1.18) for MCV-D2) (Fig 5).

There was a gradient from children living in the poorest wealth quintiles having the lowest HR for vaccination compared to children who live in their country’s richest wealth quintile, who had the highest HR for vaccination (Fig 5). Although this was observed across all vaccines, the gradient was steepest for birth doses. A similar gradient was observed according to the children’s mother’s level of formal education: children whose mothers achieved the highest level of formal education had the highest vaccination HR. No clear differences were observed in the HR for being vaccinated according to the children’s sex at birth or mother’s marital status (S7 Fig), but those whose mother’s husbands were employed had significantly higher HR for vaccination compared to children to whose mother’s husbands were unemployed.

### Association of vaccination timeliness and course completion

Vaccination delay of the first dose of each course was associated with not receiving the later doses (Table 1). For each week of delay in receiving the first dose, children had significantly lower odds of receiving the second and third doses; for example, 0.970 (0.969, 0.971) and 0.974 (0.973, 0.974) for DTP-D2 and DTP-D3, and 0.935 (0.933, 0.936) and 0.945 (0.943, 0.946) for RV-D2 and RV-D3.

**Table 1:** Association of vaccination course completion and first dose vaccination delay.

	For every week of D1 delay:	
	OR (95% CI) for receiving D2	OR (95% CI) for receiving D3
**DTP**	0.970 (0.969, 0.971)	0.974 (0.973, 0.974)
**HepB**	0.962 (0.960, 0.963)	0.961 (0.959, 0.962)
**Hib**	0.953 (0.950, 0.955)	0.951 (0.948, 0.953)
**MCV**	0.988 (0.987, 0.990)	NA
**OPV**	0.962 (0.961, 0.964)	0.960 (0.959, 0.961)
**PCV**	0.926 (0.923, 0.928)	0.933 (0.931, 0.935)
**RV**	0.935 (0.933, 0.936)	0.945 (0.943, 0.946)

The OR (95% CI) for receiving each course’s second and third doses for each week of delay in their corresponding first dose is indicated. Abbreviations: BCG, Bacillus Calmette-Guérin; 95% CI, 95% Confidence Intervals; D1/2/3, Doses 1, 2 or 3; DTP, Diphtheria-Tetanus-Pertussis; HepB, Hepatitis B vaccine; Hib, Haemophilus influenzae vaccine; MCV, Measles-Containing Vaccine; OR, Odds Ratio; OPV, Oral Polio Vaccine; PCV, Pneumococcus Vaccine; RV, Rotavirus vaccine.

## Discussion

In this study, we performed an analysis of vaccination timeliness for 24 childhood routine immunisations using recent DHS data from 54 countries. The last comprehensive analyses on vaccination timeliness, which described vaccination delay as a continuous outcome across multiple countries, were published in 2009 and 2011 [9, 10]. Here, in addition to assessing vaccination timeliness for BCG, DTP, OPV and MCV-D1, which have been largely studied [8–10], we also characterised timeliness for more recently introduced vaccinations such as: Hepatitis B, *Haemophilus influenzae type b*, Pneumococcus and Rotavirus. Furthermore, using data from several birth cohorts, vaccination delay over time was analysed. Finally, we performed a survival analysis and Cox regression to identify indicators associated with delayed vaccination [28].

Initial coverage (coverage at each vaccine’s recommended age of vaccination), and final coverage achieved, were highest for vaccines administered earlier in life (such as BCG or first doses) and lowest for second and third doses. The higher initial coverage indicates that the proportion of children receiving birth doses (such as BCG or OPV-Birth Dose) and first doses (such as DTP-D1 or OPV-D1) at the recommended ages is higher than the proportion of children who receive later doses (such as DTP-D3 or OPV-D3) at their respective recommended ages. Clark and Sanderson’s analysis over 45 low- and middle-income countries estimated that 49% of children received BCG four weeks after the recommendation, 24% received DTP-D1 two weeks after the recommendation and 12% received MCV-D1 at nine months of age (target age) [9]. Although they also measured a higher proportion of vaccination at their recommended age for initial doses, the proportions measured in this study are higher: 72.7% of children received BCG four weeks after the recommendation, 44.9% received DTP-D1 two weeks after the recommendation and 16.3% received MCV-D1 at the recommended age (nine months). Both studies were performed using DHS data, but the specific countries included varied; however, of the 45 countries included in Clark and Sanderson analysis, all but eight were also analysed in our study using a more recent DHS survey. This suggests an improvement in routine immunisation programmes achieving a higher proportion of timely vaccinations. Interestingly, although HepB-BD and OPV-BD have higher initial coverages than HepB-D1 and OPV-D1, respectively, they reach lower final coverages than their corresponding first dose. The higher final coverage of OPV-D1 and HepB-D1 could be due to these immunisations being present in many more countries than their corresponding birth doses.

In addition to a smaller proportion of children receiving second and third doses at their recommended ages, these vaccinations also had higher median vaccination delay and presented longer tails in the vaccination delay distribution. Previous studies analysing vaccination delay globally have also measured larger delays among later doses: Clark and Sanderson estimated 2.3 weeks for BCG, 2.4 weeks for DTP1, 2.7 weeks for MCV1 and 6.2 weeks for DTP3 [9], and Akmatov et al. estimated 2.1 weeks for BCG, 2.4 weeks for DTP1, 6.3 weeks for DTP3, 2.0 weeks for Polio1, 6.6 weeks for Polio3 and 4.1 weeks for MCV1 [10]. Although these studies were published in 2009 and 2011, respectively, our vaccination delay estimates are similar, which is concordant with our observation that vaccination delay has remained largely constant across birth cohorts over the last decade. We did not observe an increase in vaccination delay among children born in or after 2020 (the year that the COVID-19 pandemic started), even though several studies have reported a decrease in routine immunisation services after the COVID19 pandemic [36–38]. These initial findings indicate that the pandemic led to lower coverage but did not affect patterns of vaccination timing, i.e. the children whose routine immunisation activities were not disrupted during the pandemic and were vaccinated, did so timely. However, it is still too early to assess the impact of COVID-19 on delay vaccinations through DHS data, as only 10 surveys included here were conducted after 2020.

Nevertheless, although the median vaccination delay for all vaccines did not change substantially over time, the range in estimates moderately decreased among recent cohorts, thus reducing the mean delays observed. Interestingly, this reduction in the mean delay among recent birth cohorts was more pronounced for vaccines that had been introduced recently in the countries: Hepatitis B, Hib, PCV and RV. For most of the countries analysed here, vaccination with HepB, Hib, PCV and RV had only been introduced in the last ten years prior to the DHS survey. The larger reduction of mean vaccination delay for these vaccines could indicate an adjustment until all routine immunisation services offered Hep, Hib, PCV and RV.

Few studies have previously analysed vaccination timeliness for Hepatitis B, Hib, PCV and RV [12–14, 21]. Wariri et al. measured a median vaccination delay of 16 days for HepB-BD and 15 days for pentavalent-D1 (including HepB-D1 and Hib-D1) in The Gambia [14], which match our estimates of median delay for this country of 2 weeks for HepB-BD and 2 weeks for HepB-D1 and Hib-D1. Dejene et al. and Dirirsa et al. reported that most children in Ethiopia receive all three HepB and Hib doses within four weeks of recommendation [12, 13], and we estimated that the median delay for these vaccines in Ethiopia was between two and three weeks. Blose et al. estimated a median delay of 7.0 weeks for DTP-IPV-HiB-D1 and 7.6 weeks for HepB-D1 in South Africa [21], which is much higher than our estimates of median delay in the same country for those vaccine doses (ranging between 1 to 2 weeks). However, both studies had different study population sizes (Blose et al. comprised 500–700 children whereas here there were 3,413 surveyed children from South Africa) and recruitment (hospital versus household survey). Although some studies have characterised vaccination timeliness for PCV and RV [12, 13], they did not provide a quantitative measure but rather a categorical definition: “early”, “timely” and “delayed”. Both studies consider timely vaccination if the dose is given within four weeks after recommendation [12, 13], and they both report that most children received PCV and RV on time. This is concordant with our results, where we report median delays of two to three weeks for all doses of PCV and RV in Ethiopia.

Our estimates of median vaccination delay were mostly of one to five weeks, meaning most children are generally vaccinated within a few weeks of the recommended age and benefit from vaccination protection within an acceptable window. However, some of children were vaccinated more than six months after recommendations (up to 5% for IPV-D1, MCV-D1 or OPV-D3), which could limit the immune protection of vaccines. Additionally, children receiving first doses after the recommended age were less likely to complete vaccination courses. This suggests that children receiving first doses after the recommendation are more likely to lose contact with immunisation services, thus overall limiting the impact of vaccination. Furthermore, we found that vaccination delay varied across different countries, reaching much higher median delays in some. In Chad, we observed median delays of up to 11 weeks (IQR: 4, 30) for DTP-D3, 13 weeks for IPV-D1 in Bangladesh and Guinea, and in the Dominican Republic, median vaccination delay for RV-D1 was 45 (IQR: 43, 49). Finally, we also observed that children from deprived socioeconomic backgrounds were more likely to receive vaccinations after the recommended age.

Children living in a rural area had a higher risk of being vaccinated after the recommended age with birth and first doses than those living in an urban area. Other studies have corroborated that delayed BCG vaccination was more likely among children living in a rural area [10, 13]. This higher risk could be a result of home deliveries, as Noh et al. found that institutional deliveries were associated with timely vaccinations [39]. Caregiver’s formal education level has also been reported to be associated with higher risk of receiving delayed vaccinations [13, 15, 26].

Our study has several limitations. First, the analyses were performed on children reached by the DHS survey, which may or may not reflect the entire population. Furthermore, delay in vaccination analyses are limited to vaccinated children, who may be a different group to those not vaccinated. However, DHS surveys are designed to be representative of the national population, reaching between 5,000 to 30,000 households depending on each country [40, 41]. Additionally, among those vaccinated and reached by the DHS surveys, there were no large demographic or socio-economic differences among vaccinated children with and without vaccination date data (measured as bigger than 5% difference in cross-tabulations for demographic and socio-economic categories), except for possession of health cards, which are the main source of vaccination timing data. Second, the number of children included in each survey varies from country to country (ranging from 2,712 in Turkey to 224,218 in India), which means the overall estimates are highly driven by the most populated countries, such as India and Nigeria. Furthermore, most of the countries included in the analyses belong to the WHO African region, which would also bias the representativeness of this region versus others. However, we also present our results broken down by country and, in our Cox regression model, we accounted for a country-random effect. Although there is high variability in the coverage per week and delay estimates between countries, the general trend by which later doses reach lower coverages and experience the largest delays is maintained. Third, we used the most recent available vaccine schedules available in the WHO website as the reference for recommended age of vaccination, which may not reflect the recommended age of vaccination on the year(s) the surveys were conducted in each country if those have changed over time. There is a high variability between the years of each country’s most recent DHS survey (from 2011 for Ethiopia to 2022 for Cambodia, Kenya and the Philippines), meaning the age recommendations used in the analyses may be closer to the recommendations in place during the DHS survey in some countries than in others. However, changes to the vaccination schedule are rare, and most countries’ recommendations were very similar to WHO’s. Fourth, although most vaccination age recommendations are similar across countries, there are still several differences across countries’ vaccination schedules. This could limit the comparability of results, yet vaccination delay estimates for all children were calculated using the recommended age for their own country. Finally, our assumption around the day of birth implies that all coverage per week of age and delay estimates have a +/- two-week time window of error. Because of this assumption, we might have overestimated vaccination delays by two weeks, but it is also possible that delay calculations were underestimated by the same amount of time.

In this study, important delays in vaccination have been reported, which suggest a substantial proportion of children are not immunized as early as possible, limiting the protection conferred by vaccines. This could reduce the overall impact of vaccination activities, especially among populations from socioeconomically deprived areas, who are more likely to receive delayed vaccinations. For instance, as maternal antibodies for measles decay around nine months of life [42, 43], children between the ages of 8 and 12 months are most vulnerable. Thus, large delays in measles vaccination (usually administered at 9 months) could leave children unprotected.

The Immunisation Agenda 2023 strategy aims to “extend the benefits of vaccines to everyone, everywhere”. This study reflects that analysing vaccination timeliness alongside coverage is also key to fully assess the performance and impact of vaccination programmes. These analyses on timing of vaccination could better inform whether vaccination reminders are needed, when they should be given to population to minimise delays in vaccination and avoid leaving children unprotected, and which populations should be specifically targeted. For example, large vaccination delays are observed in Chad for all immunisations, suggesting routine vaccination programmes could benefit from general reminders when follow-up doses are due. On the other hand, Bangladesh presents a high vaccination delay only for IPV-D1. Since IPV-D1 was introduced only three years before the latest DHS survey was conducted in Bangladesh, these results could indicate that there is still some adjustment time needed until IPV-D1 reaches the same uptake as other immunisations. Finally, they could also help in informing future vaccination strategies, such as modifications to vaccination schedules that better optimise uptake or vaccine response. As new vaccines are developed and introduced in routine immunisation programmes [3], it is important to consider how to fit these new vaccines into vaccination schedules.

## Supporting information

S1 Fig Recommended age of vaccination for each vaccine and dose in each country. The recommended age for vaccination with each vaccine and dose is shown as the difference (in weeks) between each country’s vaccination age recommendation and the WHO recommendation (26). A white dot in the x = 0 line indicates the country’s vaccination recommendation is identical to the WHO’s (top row) for that vaccine and dose. A negative difference (in green) indicates the country’s recommended age of vaccination is smaller than the WHO’s, while a positive difference (in red) indicates the country’s recommended age is bigger than the WHO’s. WHO recommended vaccination age is considered at [26]: birth for birth doses (BCG, HepB-BD, OPV-BD); 6 weeks of age for DTP-D1, HepB-D1, Hib-D1, IPV-D1, OPV-D1, PCV-D1 and RV-D1; 10 weeks of age for DTP-D2, HepB-D2, Hib-D2, OPV-D2, PCV-D2 and RV-D2; 14 weeks of age for DTP-D3, HepB-D3, Hib-D3, OPV-D3, PCV-D3 and RV-D3; 39 weeks for MCV-D1 and 65 weeks for MCV-D2. Abbreviations: BCG, Bacillus Calmette-Guérin; BD, Birth Dose; D1/2/3, Doses 1, 2 or 3; DTP, Diphtheria-Tetanus-Pertussis; HepB, Hepatitis B vaccine; Hib, Haemophilus influenzae vaccine; IPV, Inactivated Polio Vaccine; MCV, Measles-Containing Vaccine; OPV, Oral Polio Vaccine; PCV, Pneumococcus Vaccine; RV, Rotavirus vaccine; WHO, World Health Organisation.
(TIFF)

S2 Fig Percentage of children from each country among all included in the analyses. The percentage of surveyed children from each country among all those captured in all surveys (n = 743,694, S4 Table) is indicated in a colour scale. Note: the colour scale is log10 transformed. The map base layer was obtained from the R package “rnaturalearth” [44] available at https://cran.r-project.org/web/packages/rnaturalearth/index.html.
(TIFF)

S3 Fig Demographic and socioeconomic characteristics of children with and without (A) vaccination data or (B) vaccination age data. The percentage of children who had vaccination data (A) or vaccination age data (B) is shown on x-axis versus the percentage of children who did not vaccination data (A) or vaccination age data (B) (on y-axis) among the different subcategories (e.g. male versus female) of each demographic/socioeconomic indicator is represented.
(TIFF)

S4 Fig Vaccination coverage per week of age for each country. Coverage per week of age is calculated as the cumulative proportion of children vaccinated at each week of age. All countries pulled coverage estimates (top row) are shown for the WHO recommended vaccination week of age for each vaccine and dose; two, four, six, and eight weeks later; four and six months later; and at five years of age (final coverage). Each country’s coverage estimates are shown for that country’s recommended vaccination week of age for each vaccine and dose; two, four, six, and eight weeks later; four and six months later; and at five years of age (final coverage). WHO recommended vaccination age is considered at [1]: the first week of age for birth doses (BCG, HepB-BD, OPV-BD); 6 weeks of age for DTP-D1, HepB-D1, Hib-D1, IPV-D1, OPV-D1, PCV-D1 and RV-D1; 10 weeks of age for DTP-D2, HepB-D2, Hib-D2, OPV-D2, PCV-D2 and RV-D2; 14 weeks of age for DTP-D3, HepB-D3, Hib-D3, OPV-D3, PCV-D3 and RV-D3; 39 weeks for MCV-D1 and 65 weeks for MCV-D2. Abbreviations: BCG, Bacillus Calmette-Guérin; BD, Birth Dose; D1/2/3, Doses 1, 2 or 3; DTP, Diphtheria-Tetanus-Pertussis; HepB, Hepatitis B vaccine; Hib, Haemophilus influenzae vaccine; IPV, Inactivated Polio Vaccine; MCV, Measles-Containing Vaccine; OPV, Oral Polio Vaccine; PCV, Pneumococcal Vaccine; RV, Rotavirus vaccine; WHO, World Health Organisation.
(TIFF)

S5 Fig Vaccination coverage per week of age for each country. Coverage per week of age is calculated as the cumulative proportion of children vaccinated at each week of age. All countries pulled coverage estimates (top row) are shown for the WHO recommended vaccination week of age for each vaccine and dose; two, four, six, and eight weeks later; four and six months later; and at five years of age (final coverage). Each country’s coverage estimates are shown for that country’s recommended vaccination week of age for each vaccine and dose; two, four, six, and eight weeks later; four and six months later; and at five years of age (final coverage). WHO recommended vaccination age is considered at [1]: the first week of age for birth doses (BCG, HepB-BD, OPV-BD); 6 weeks of age for DTP-D1, HepB-D1, Hib-D1, IPV-D1, OPV-D1, PCV-D1 and RV-D1; 10 weeks of age for DTP-D2, HepB-D2, Hib-D2, OPV-D2, PCV-D2 and RV-D2; 14 weeks of age for DTP-D3, HepB-D3, Hib-D3, OPV-D3, PCV-D3 and RV-D3; 39 weeks for MCV-D1 and 65 weeks for MCV-D2. Abbreviations: BCG, Bacillus Calmette-Guérin; BD, Birth Dose; D1/2/3, Doses 1, 2 or 3; DTP, Diphtheria-Tetanus-Pertussis; HepB, Hepatitis B vaccine; Hib, Haemophilus influenzae vaccine; IPV, Inactivated Polio Vaccine; MCV, Measles-Containing Vaccine; OPV, Oral Polio Vaccine; PCV, Pneumococcal Vaccine; RV, Rotavirus vaccine; WHO, World Health Organisation.
(TIFF)

S6 Fig Vaccination coverage per week of age for each country. Coverage per week of age is calculated as the cumulative proportion of children vaccinated at each week of age. All countries pulled coverage estimates (top row) are shown for the WHO recommended vaccination week of age for each vaccine and dose; two, four, six, and eight weeks later; four and six months later; and at five years of age (final coverage). Each country’s coverage estimates are shown for that country’s recommended vaccination week of age for each vaccine and dose; two, four, six, and eight weeks later; four and six months later; and at five years of age (final coverage). WHO recommended vaccination age is considered at [1]: the first week of age for birth doses (BCG, HepB-BD, OPV-BD); 6 weeks of age for DTP-D1, HepB-D1, Hib-D1, IPV-D1, OPV-D1, PCV-D1 and RV-D1; 10 weeks of age for DTP-D2, HepB-D2, Hib-D2, OPV-D2, PCV-D2 and RV-D2; 14 weeks of age for DTP-D3, HepB-D3, Hib-D3, OPV-D3, PCV-D3 and RV-D3; 39 weeks for MCV-D1 and 65 weeks for MCV-D2. Abbreviations: BCG, Bacillus Calmette-Guérin; BD, Birth Dose; D1/2/3, Doses 1, 2 or 3; DTP, Diphtheria-Tetanus-Pertussis; HepB, Hepatitis B vaccine; Hib, Haemophilus influenzae vaccine; IPV, Inactivated Polio Vaccine; MCV, Measles-Containing Vaccine; OPV, Oral Polio Vaccine; PCV, Pneumococcal Vaccine; RV, Rotavirus vaccine; WHO, World Health Organisation.
(TIFF)

S7 Fig Multivariate analysis of demographic and socioeconomic indicators associated with vaccination delay. Hazard Ratio (and 95% Confidence intervals) for vaccination with each vaccine according to each covariate is represented on the x-axis. Vaccines (on the y-axis) are ordered according to WHO recommended vaccination age [1]. Abbreviations: BCG, Bacillus Calmette-Guérin; BD, Birth Dose; D1/2/3, Doses 1, 2 or 3; DTP, Diphtheria-Tetanus-Pertussis; HepB, Hepatitis B vaccine; Hib, Haemophilus influenzae vaccine; IPV, Inactivated Polio Vaccine; MCV, Measles-Containing Vaccine; OPV, Oral Polio Vaccine; PCV, Pneumococcus Vaccine; RV, Rotavirus vaccine; WHO, World Health Organisation.
(TIFF)

S1 Table Demographic and socioeconomic indicators. Information for each child was extracted from the DHS survey, as coded on the second column, and recoded (third column) when number of categories was large. Abbreviations: DHS, Demographic and Health Surveys.
(PDF)

S2 Table Year of vaccine introduction and DHS most recent survey for each country. Year of introduction for each vaccine in each country is indicated [26], alongside the DHS survey-year included in the analysis (in parenthesis). All countries were considered to have introduced BCG, all three doses of DTP and OPV, and the first dose of MCV by the time of the survey. Abbreviations: BCG, Bacillus Calmette-Guérin; BD, Birth Dose; D1/2/3, Doses 1, 2 or 3; DHS, Demographic and Health Surveys; DTP, Diphtheria-Tetanus-Pertussis; HepB, Hepatitis B vaccine; Hib, Haemophilus influenzae vaccine; IPV, Inactivated Polio Vaccine; MCV, Measles-Containing Vaccine; OPV, Oral Polio Vaccine; PCV, Pneumococcal Vaccine; RV, Rotavirus vaccine.
(PDF)

S3 Table Variables selected during model selection. For each vaccine, we constructed a multivariable Cox regression model using stepwise-variable selection methods: “selectCox” [31] (backwards selection) and “stepwiseCox” [32] (forward selection). The variables marked with an “x” are the ones selected by each method for each vaccine’s multivariable model. Abbreviations: BCG, Bacillus Calmette-Guérin; BD, Birth Dose; D1/2/3, Doses 1, 2 or 3; DTP, Diphtheria-Tetanus-Pertussis; HepB, Hepatitis B vaccine; Hib, Haemophilus influenzae vaccine; IPV, Inactivated Polio Vaccine; IQR, Interquartile Range; MCV, Measles-Containing Vaccine; OPV, Oral Polio Vaccine; PCV, Pneumococcal Vaccine; RV, Rotavirus vaccine.
(PDF)

S4 Table Number of children captured in the surveys, per country and per birth cohort. Total number of live children captured in all the surveys, number of children captured in each country’s survey, and number of children born in each calendar year among all countries’ surveys. For each country, year of survey in indicated. Median age at time of survey is shown in months. Abbreviations: IQR, Interquartile Range.
(PDF)

S5 Table Number of countries and children with data available for each vaccine. Proportion of countries with/without the vaccine introduced was estimated as the number of countries in which the vaccine had / had not been introduced by the time of the survey among all 54 included in the analysis. Percentage of children from countries in which each vaccine had been introduced by the time of the survey was calculated over the total number of alive children captured in the surveys (n = 743,694, S4 Table). Proportion of children with vaccination data for each vaccine was estimated over all those children from countries in which the vaccine had been introduced. Proportion of children with at least one year buffer time (time between recommended date of vaccination and survey was ≥1 year) was estimated over all of those with vaccination data. Percentage of vaccinated children was calculated over all of those with vaccination data with one year buffer time. Percentage of children with vaccination age (date) data was estimated over all those vaccinated. Abbreviations: BCG, Bacillus Calmette-Guérin; BD, Birth Dose; D1/2/3, Doses 1, 2 or 3; DTP, Diphtheria-Tetanus-Pertussis; HepB, Hepatitis B vaccine; Hib, Haemophilus influenzae vaccine; IPV, Inactivated Polio Vaccine; MCV, Measles-Containing Vaccine; OPV, Oral Polio Vaccine; PCV, Pneumococcal Vaccine; RV, Rotavirus vaccine.
(PDF)

S6 Table Median vaccination delay (and IQR) per country. Vaccination delay is defined as the difference between age at vaccination and recommended age for vaccination, in weeks. For each country, year of survey in indicated. Abbreviations: BCG, Bacillus Calmette-Guérin; BD, Birth Dose; D1/2/3, Doses 1, 2 or 3; DTP, Diphtheria-Tetanus-Pertussis; HepB, Hepatitis B vaccine; Hib, Haemophilus influenzae vaccine; IPV, Inactivated Polio Vaccine; IQR, Interquartile Range; MCV, Measles-Containing Vaccine; OPV, Oral Polio Vaccine; PCV, Pneumococcal Vaccine; RV, Rotavirus vaccine.
(PDF)
