# Supplementary figures and images for: Timeliness of 24 childhood immunisations and evolution of vaccination delay: Analysis of data from 54 low- and middle-income countries
Source: PLOS Glob Public Health. 2024 Nov 26;4(11):e0003749. doi: 10.1371/journal.pgph.0003749 (PMC11593752; doi:10.1371/journal.pgph.0003749)

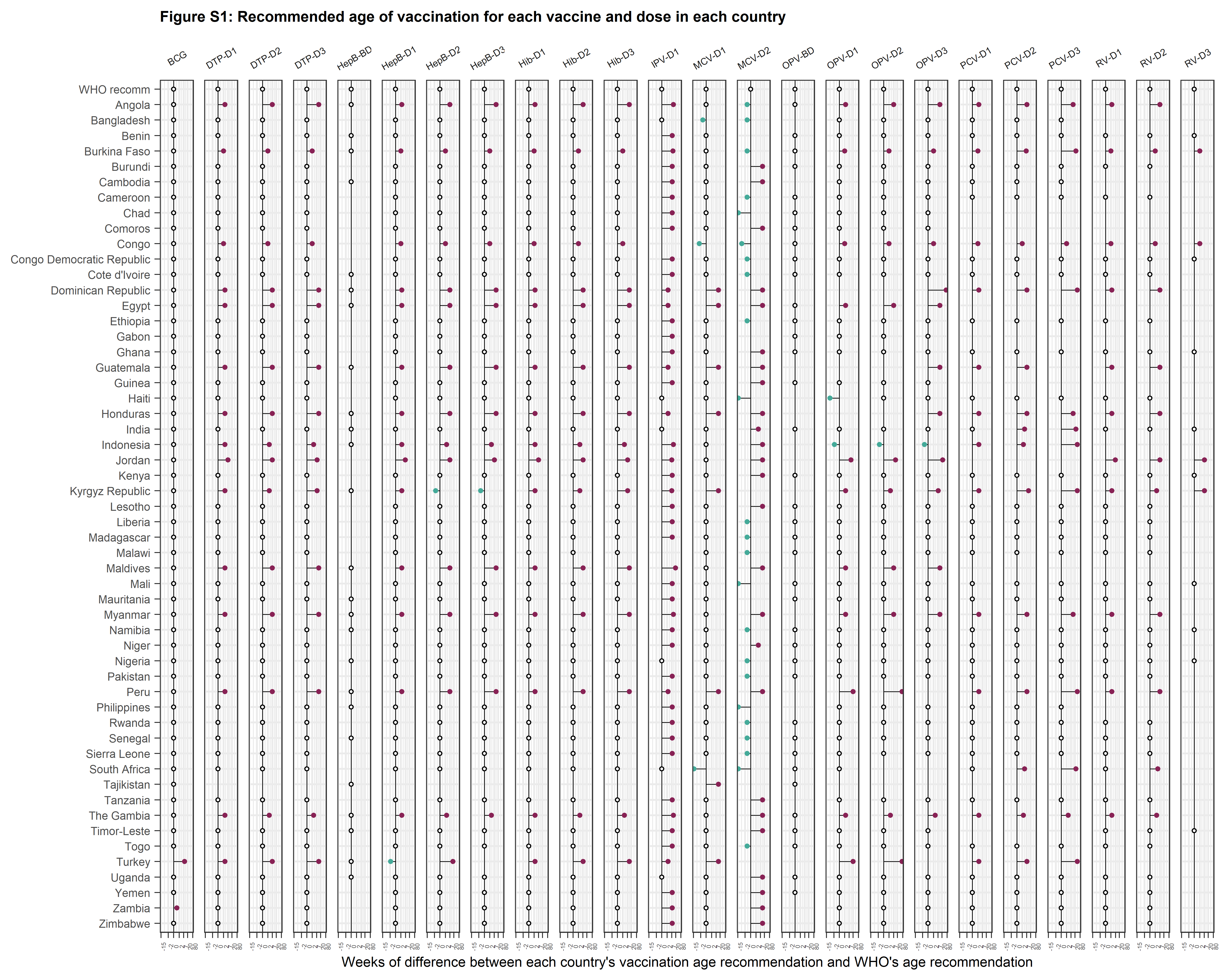

Supplement: S1 Fig — The recommended age for vaccination with each vaccine and dose is shown as the difference (in weeks) between each country’s vaccination age recommendation and the WHO recommendation (26). A white dot in the x = 0 line indicates the country’s vaccination recommendation is identical to the WHO’s (top row) for that vaccine and dose. A negative difference (in green) indicates the country’s recommended age of vaccination is smaller than the WHO’s, while a positive difference (in red) indicates the country’s recommended age is bigger than the WHO’s. WHO recommended vaccination age is considered at [26]: birth for birth doses (BCG, HepB-BD, OPV-BD); 6 weeks of age for DTP-D1, HepB-D1, Hib-D1, IPV-D1, OPV-D1, PCV-D1 and RV-D1; 10 weeks of age for DTP-D2, HepB-D2, Hib-D2, OPV-D2, PCV-D2 and RV-D2; 14 weeks of age for DTP-D3, HepB-D3, Hib-D3, OPV-D3, PCV-D3 and RV-D3; 39 weeks for MCV-D1 and 65 weeks for MCV-D2. Abbreviations: BCG, Bacillus Calmette-Guérin; BD, Birth Dose; D1/2/3, Doses 1, 2 or 3; DTP, Diphtheria-Tetanus-Pertussis; HepB, Hepatitis B vaccine; Hib, Haemophilus influenzae vaccine; IPV, Inactivated Polio Vaccine; MCV, Measles-Containing Vaccine; OPV, Oral Polio Vaccine; PCV, Pneumococcus Vaccine; RV, Rotavirus vaccine; WHO, World Health Organisation. (TIFF) [file pgph.0003749.s001.tiff]

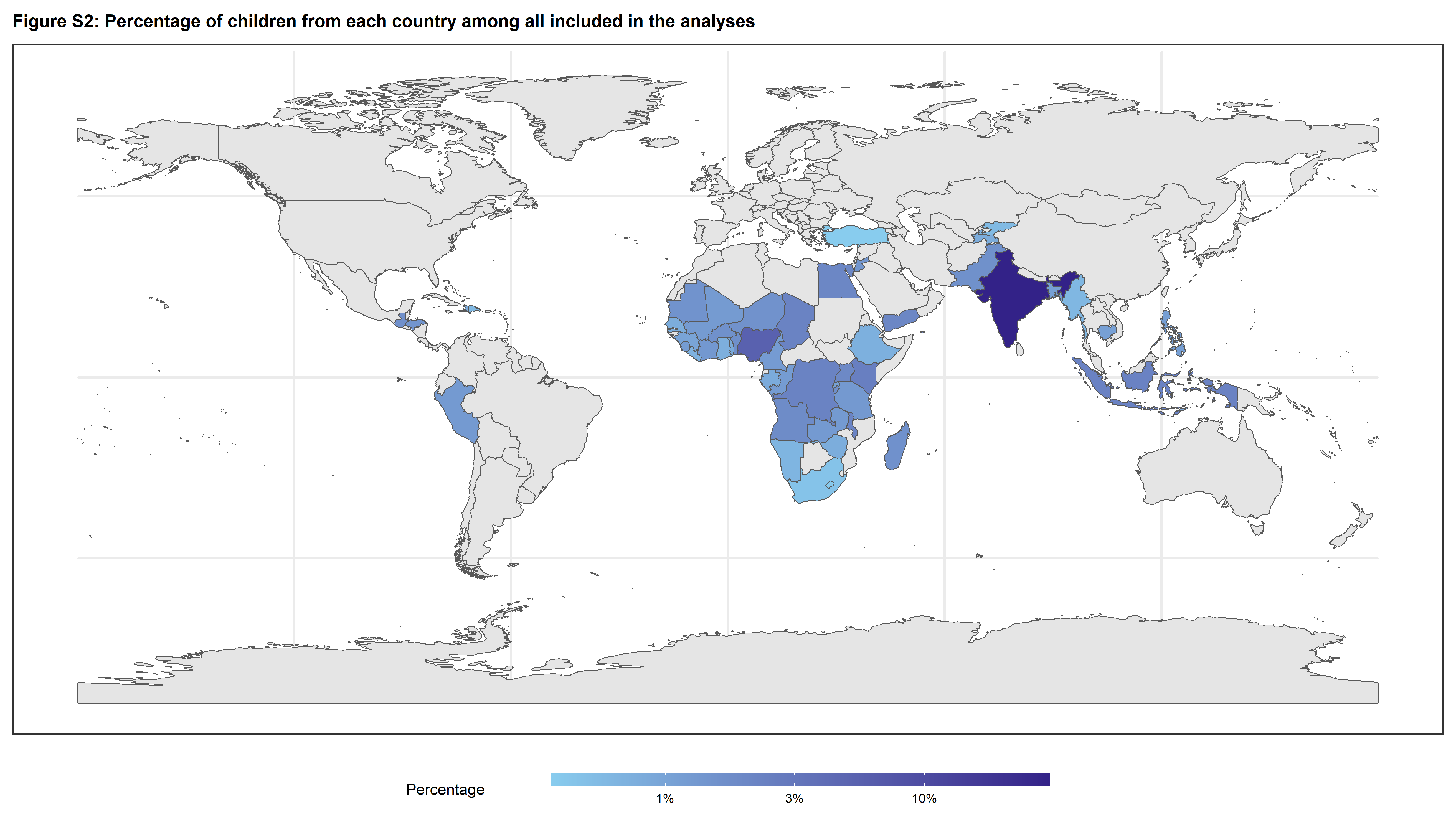

Supplement: S2 Fig — The percentage of surveyed children from each country among all those captured in all surveys (n = 743,694, S4 Table) is indicated in a colour scale. Note: the colour scale is log10 transformed. The map base layer was obtained from the R package “rnaturalearth” [44] available at https://cran.r-project.org/web/packages/rnaturalearth/index.html. (TIFF) [file pgph.0003749.s002.tiff]

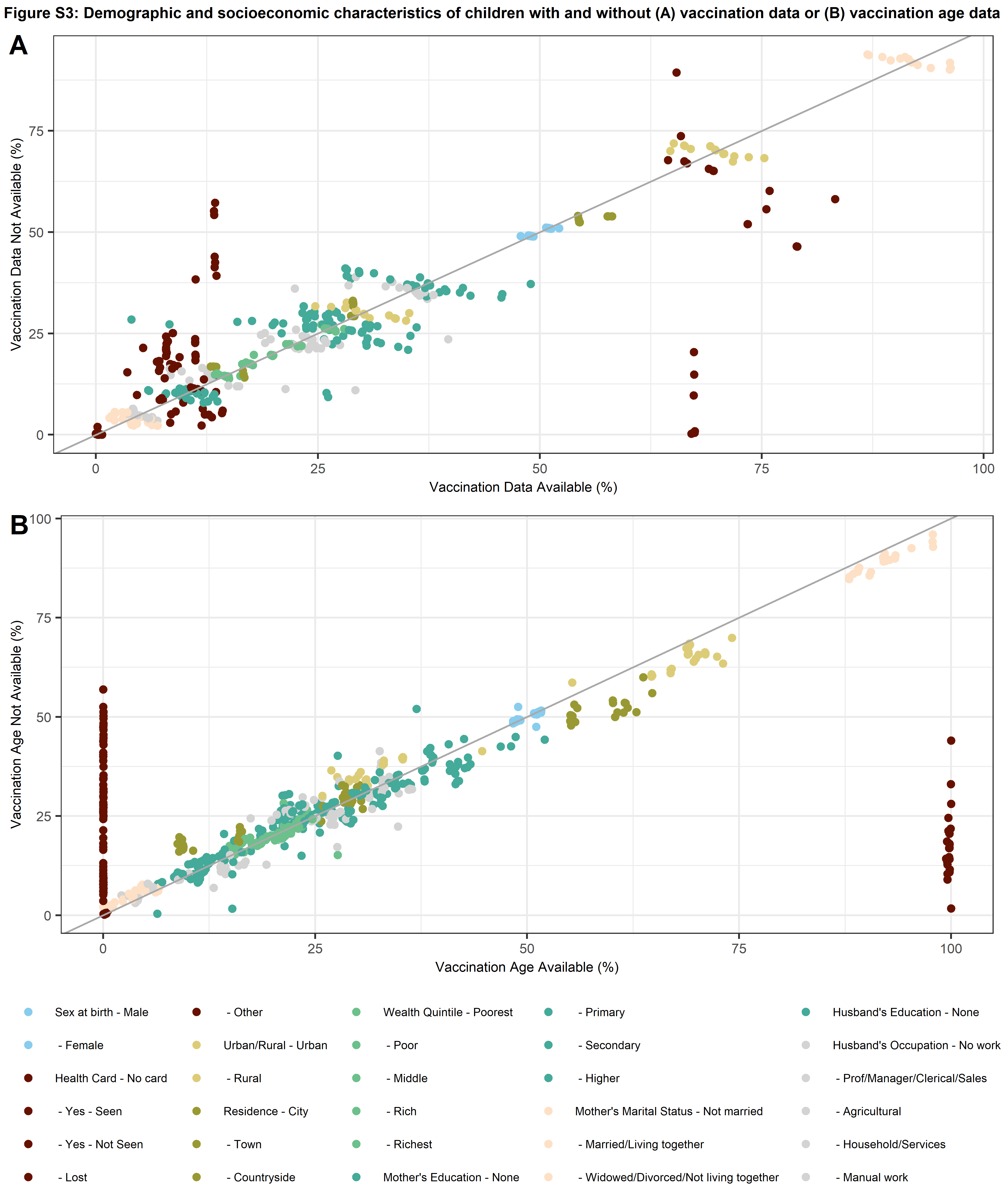

Supplement: S3 Fig — Demographic and socioeconomic characteristics of children with and without (A) vaccination data or (B) vaccination age data. The percentage of children who had vaccination data (A) or vaccination age data (B) is shown on x-axis versus the percentage of children who did not vaccination data (A) or vaccination age data (B) (on y-axis) among the different subcategories (e.g. male versus female) of each demographic/socioeconomic indicator is represented. (TIFF) [file pgph.0003749.s003.tiff]

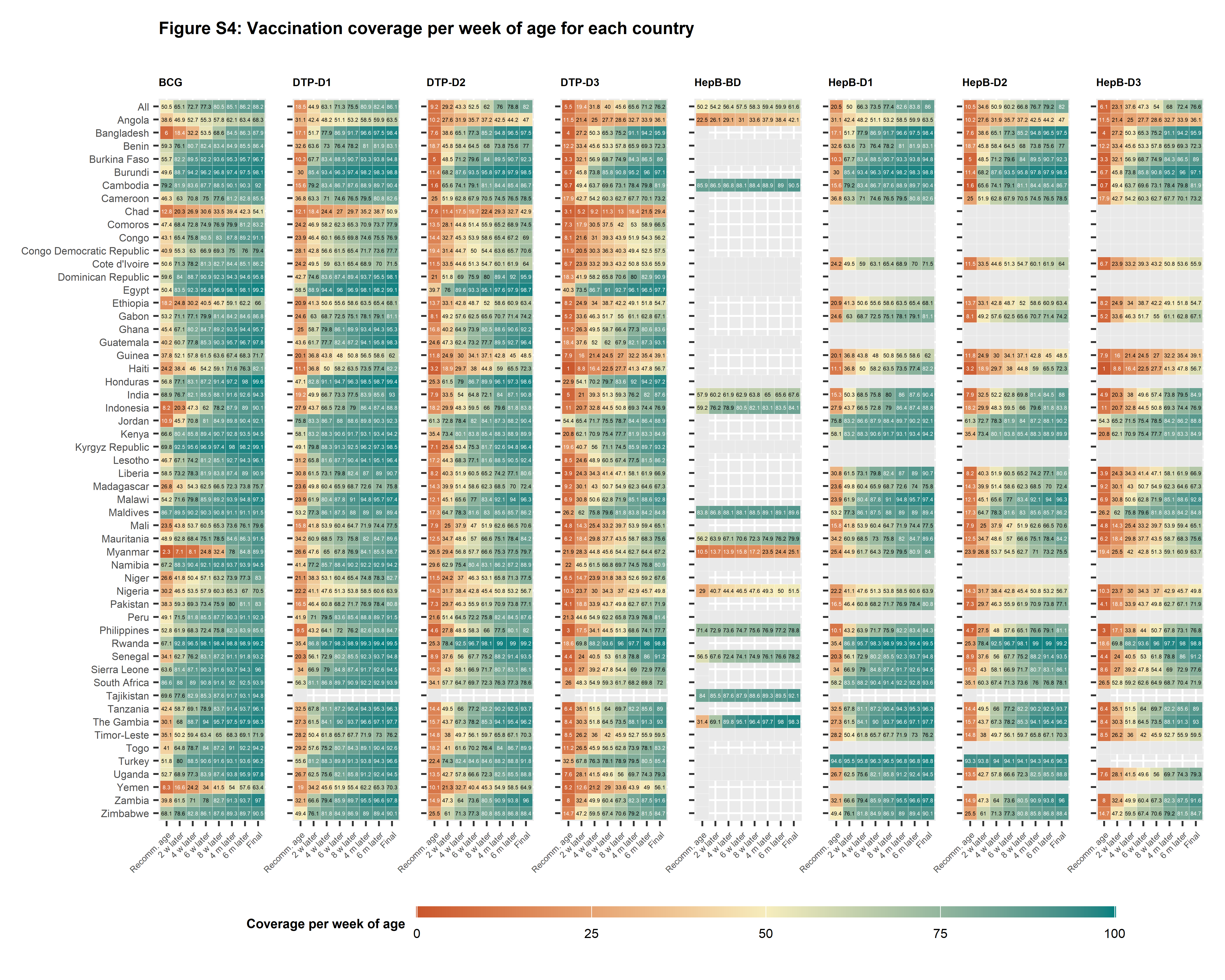

Supplement: S4 Fig — Coverage per week of age is calculated as the cumulative proportion of children vaccinated at each week of age. All countries pulled coverage estimates (top row) are shown for the WHO recommended vaccination week of age for each vaccine and dose; two, four, six, and eight weeks later; four and six months later; and at five years of age (final coverage). Each country’s coverage estimates are shown for that country’s recommended vaccination week of age for each vaccine and dose; two, four, six, and eight weeks later; four and six months later; and at five years of age (final coverage). WHO recommended vaccination age is considered at [1]: the first week of age for birth doses (BCG, HepB-BD, OPV-BD); 6 weeks of age for DTP-D1, HepB-D1, Hib-D1, IPV-D1, OPV-D1, PCV-D1 and RV-D1; 10 weeks of age for DTP-D2, HepB-D2, Hib-D2, OPV-D2, PCV-D2 and RV-D2; 14 weeks of age for DTP-D3, HepB-D3, Hib-D3, OPV-D3, PCV-D3 and RV-D3; 39 weeks for MCV-D1 and 65 weeks for MCV-D2. Abbreviations: BCG, Bacillus Calmette-Guérin; BD, Birth Dose; D1/2/3, Doses 1, 2 or 3; DTP, Diphtheria-Tetanus-Pertussis; HepB, Hepatitis B vaccine; Hib, Haemophilus influenzae vaccine; IPV, Inactivated Polio Vaccine; MCV, Measles-Containing Vaccine; OPV, Oral Polio Vaccine; PCV, Pneumococcal Vaccine; RV, Rotavirus vaccine; WHO, World Health Organisation. (TIFF) [file pgph.0003749.s004.tiff]

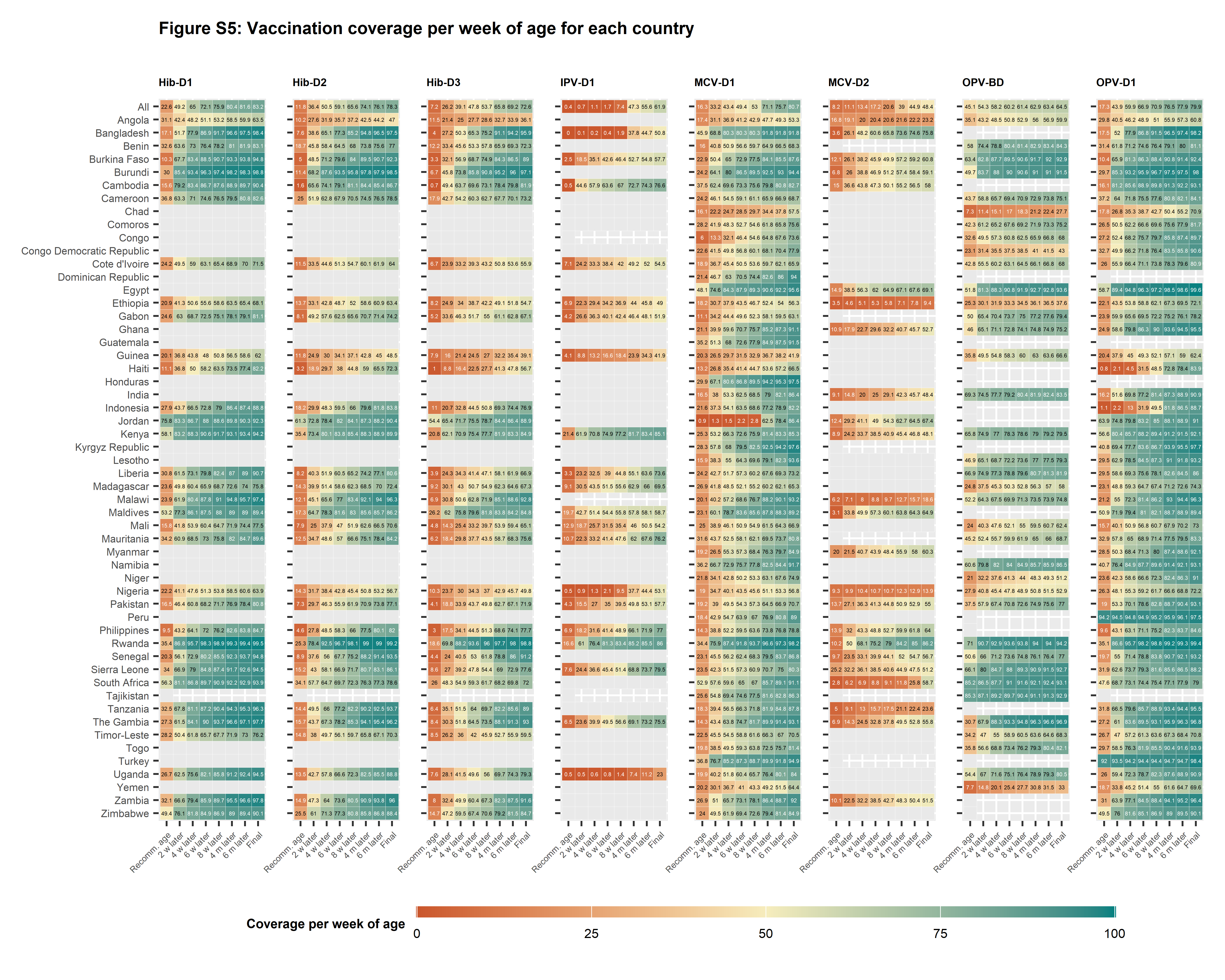

Supplement: S5 Fig — Coverage per week of age is calculated as the cumulative proportion of children vaccinated at each week of age. All countries pulled coverage estimates (top row) are shown for the WHO recommended vaccination week of age for each vaccine and dose; two, four, six, and eight weeks later; four and six months later; and at five years of age (final coverage). Each country’s coverage estimates are shown for that country’s recommended vaccination week of age for each vaccine and dose; two, four, six, and eight weeks later; four and six months later; and at five years of age (final coverage). WHO recommended vaccination age is considered at [1]: the first week of age for birth doses (BCG, HepB-BD, OPV-BD); 6 weeks of age for DTP-D1, HepB-D1, Hib-D1, IPV-D1, OPV-D1, PCV-D1 and RV-D1; 10 weeks of age for DTP-D2, HepB-D2, Hib-D2, OPV-D2, PCV-D2 and RV-D2; 14 weeks of age for DTP-D3, HepB-D3, Hib-D3, OPV-D3, PCV-D3 and RV-D3; 39 weeks for MCV-D1 and 65 weeks for MCV-D2. Abbreviations: BCG, Bacillus Calmette-Guérin; BD, Birth Dose; D1/2/3, Doses 1, 2 or 3; DTP, Diphtheria-Tetanus-Pertussis; HepB, Hepatitis B vaccine; Hib, Haemophilus influenzae vaccine; IPV, Inactivated Polio Vaccine; MCV, Measles-Containing Vaccine; OPV, Oral Polio Vaccine; PCV, Pneumococcal Vaccine; RV, Rotavirus vaccine; WHO, World Health Organisation. (TIFF) [file pgph.0003749.s005.tiff]

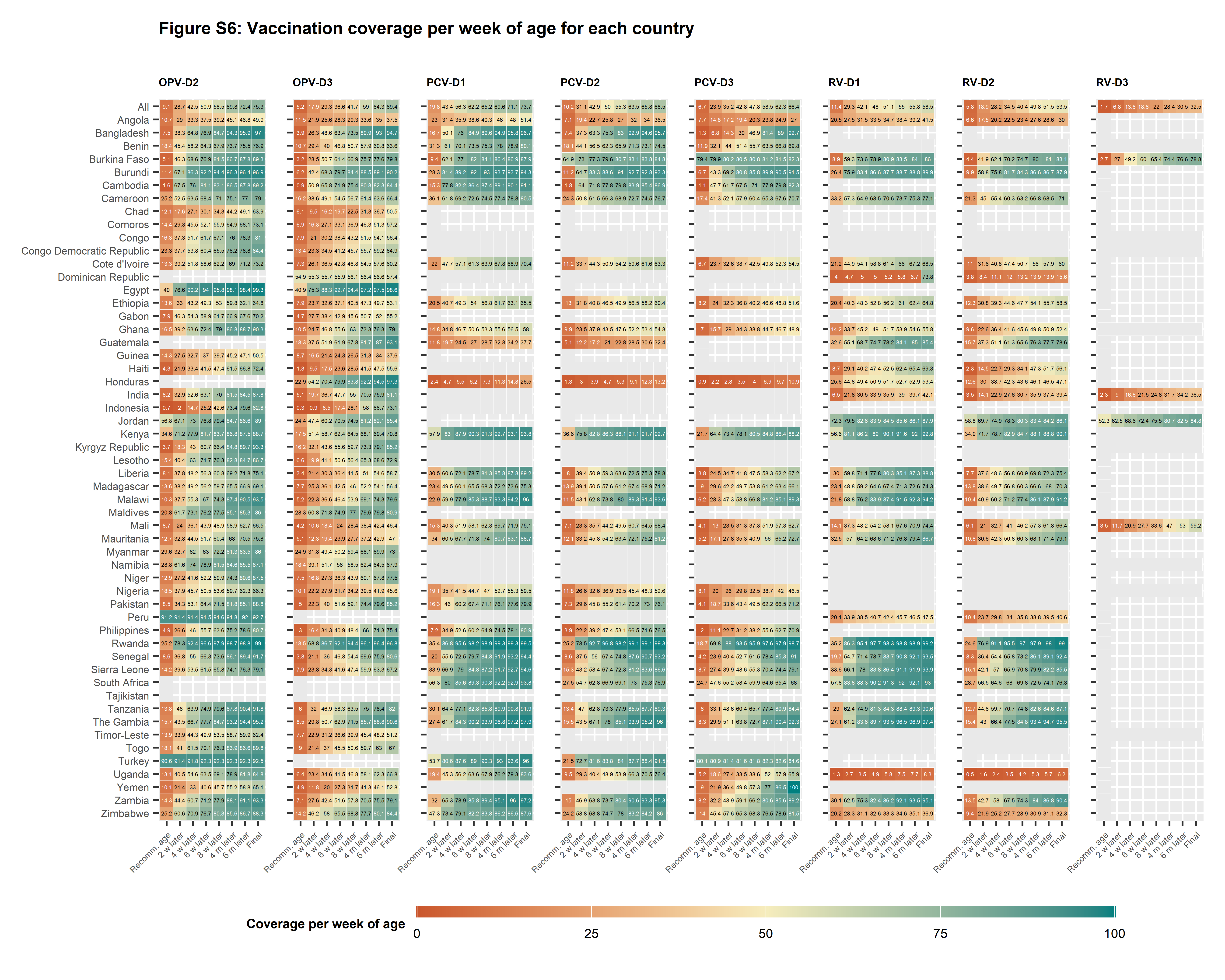

Supplement: S6 Fig — Coverage per week of age is calculated as the cumulative proportion of children vaccinated at each week of age. All countries pulled coverage estimates (top row) are shown for the WHO recommended vaccination week of age for each vaccine and dose; two, four, six, and eight weeks later; four and six months later; and at five years of age (final coverage). Each country’s coverage estimates are shown for that country’s recommended vaccination week of age for each vaccine and dose; two, four, six, and eight weeks later; four and six months later; and at five years of age (final coverage). WHO recommended vaccination age is considered at [1]: the first week of age for birth doses (BCG, HepB-BD, OPV-BD); 6 weeks of age for DTP-D1, HepB-D1, Hib-D1, IPV-D1, OPV-D1, PCV-D1 and RV-D1; 10 weeks of age for DTP-D2, HepB-D2, Hib-D2, OPV-D2, PCV-D2 and RV-D2; 14 weeks of age for DTP-D3, HepB-D3, Hib-D3, OPV-D3, PCV-D3 and RV-D3; 39 weeks for MCV-D1 and 65 weeks for MCV-D2. Abbreviations: BCG, Bacillus Calmette-Guérin; BD, Birth Dose; D1/2/3, Doses 1, 2 or 3; DTP, Diphtheria-Tetanus-Pertussis; HepB, Hepatitis B vaccine; Hib, Haemophilus influenzae vaccine; IPV, Inactivated Polio Vaccine; MCV, Measles-Containing Vaccine; OPV, Oral Polio Vaccine; PCV, Pneumococcal Vaccine; RV, Rotavirus vaccine; WHO, World Health Organisation. (TIFF) [file pgph.0003749.s006.tiff]

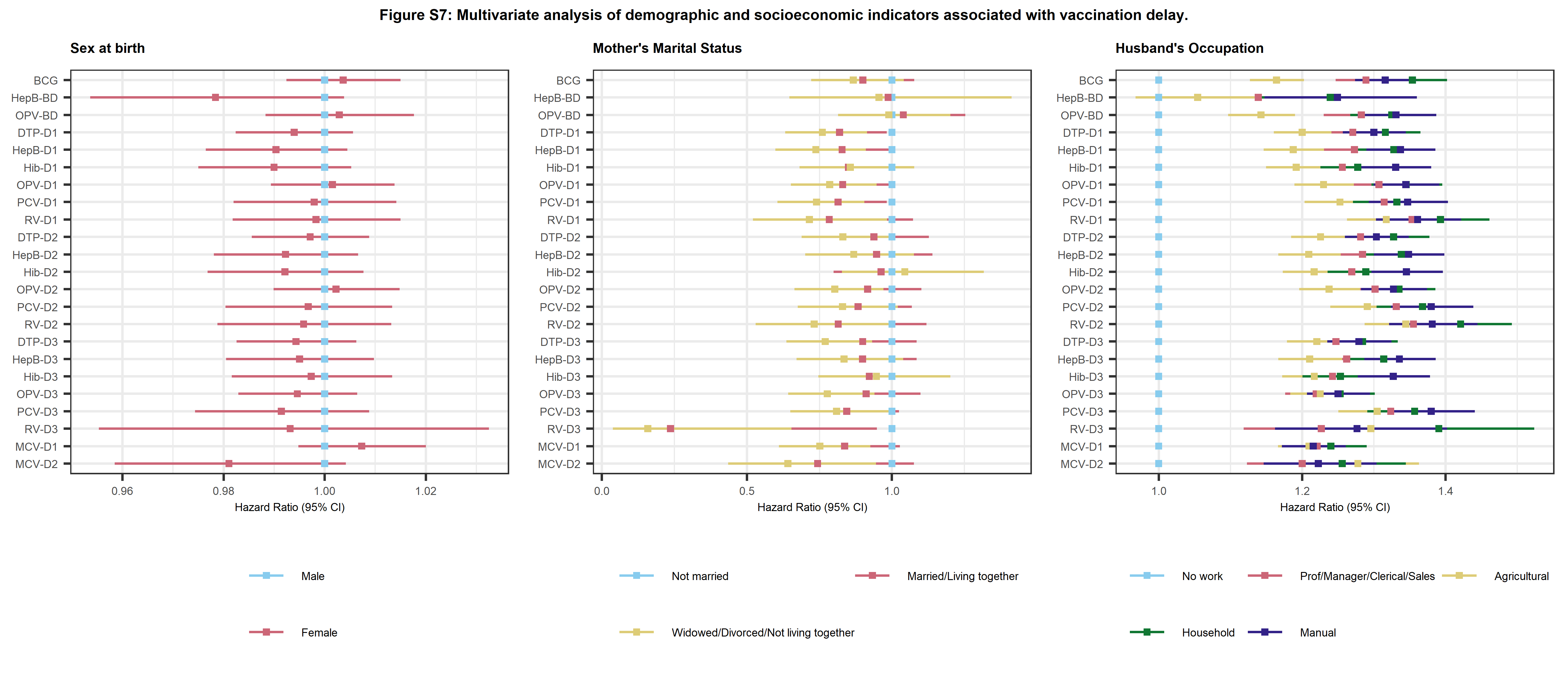

Supplement: S7 Fig — Hazard Ratio (and 95% Confidence intervals) for vaccination with each vaccine according to each covariate is represented on the x-axis. Vaccines (on the y-axis) are ordered according to WHO recommended vaccination age [1]. Abbreviations: BCG, Bacillus Calmette-Guérin; BD, Birth Dose; D1/2/3, Doses 1, 2 or 3; DTP, Diphtheria-Tetanus-Pertussis; HepB, Hepatitis B vaccine; Hib, Haemophilus influenzae vaccine; IPV, Inactivated Polio Vaccine; MCV, Measles-Containing Vaccine; OPV, Oral Polio Vaccine; PCV, Pneumococcus Vaccine; RV, Rotavirus vaccine; WHO, World Health Organisation. (TIFF) [file pgph.0003749.s007.tiff]
