# Supplementary material for: Timeliness of 24 childhood immunisations and evolution of vaccination delay: Analysis of data from 54 low- and middle-income countries
Source: PLOS Glob Public Health. 2024 Nov 26;4(11):e0003749. doi: 10.1371/journal.pgph.0003749 (PMC11593752; doi:10.1371/journal.pgph.0003749)
Supplement: S1 Table — Information for each child was extracted from the DHS survey, as coded on the second column, and recoded (third column) when number of categories was large. Abbreviations: DHS, Demographic and Health Surveys. (PDF) [file pgph.0003749.s008.pdf]

Table S1: Demographic and socioeconomic indicators.

Demographic / Socioeconomic indicators	Information recorded in the DHS questionnaire	Recoded
Sex at birth	1 – Male<br>2 – Female	NO
Possession of a health card	0 – No health card<br>1 – Has a health card, and seen<br>2 – Has a health card, not seen<br>3 – Lost health card<br>4 – Other	NO
Place of residence	1 – Urban<br>2 – Rural	NO
Place of residence	1 – City<br>2 – Town<br>3 – Countryside	NO
Household wealth quintile	1 – Poorest quintile<br>2 – Poorer quintile<br>3 – Middle quintile<br>4 – Richer quintile<br>5 – Richest quintile	NO
Children's caregivers' (Children's mother's) level of formal education	0 – None<br>1 – Primary education<br>2 – Secondary education<br>3 – Higher education	NO
Children's caregivers' (Children's mother's husband's) occupation	0 – Unemployed<br>1 – Professional / Managerial<br>2 – Clerical<br>3 – Sales<br>4 – Agricultural, self-employed<br>5 – Agricultural, employed<br>6 – Household<br>7 – Services<br>8 – Skilled manual work<br>9 – Unskilled manual work	0 – Unemployed<br>1 – Professional/Managerial / Clerical / Sales (1, 2 or 3)<br>2 – Agricultural (4 or 5)<br>3 – Household / Services (6 or 7)<br>4 – Manual work (8 or 9)
Children's mother's marital status	0 – Not married<br>1 – Married<br>2 – Living together<br>3 – Widowed<br>4 – Divorced<br>5 – Not living together	0 – Not married<br>1 – Married / Living together (1 or 2)<br>2 – Widowed / Divorced / Not living together (3, 4 or 5)
