# Supplementary material for: Timeliness of 24 childhood immunisations and evolution of vaccination delay: Analysis of data from 54 low- and middle-income countries
Source: PLOS Glob Public Health. 2024 Nov 26;4(11):e0003749. doi: 10.1371/journal.pgph.0003749 (PMC11593752; doi:10.1371/journal.pgph.0003749)
Supplement: S2 Table — Year of introduction for each vaccine in each country is indicated [26], alongside the DHS survey-year included in the analysis (in parenthesis). All countries were considered to have introduced BCG, all three doses of DTP and OPV, and the first dose of MCV by the time of the survey. Abbreviations: BCG, Bacillus Calmette-Guérin; BD, Birth Dose; D1/2/3, Doses 1, 2 or 3; DHS, Demographic and Health Surveys; DTP, Diphtheria-Tetanus-Pertussis; HepB, Hepatitis B vaccine; Hib, Haemophilus influenzae vaccine; IPV, Inactivated Polio Vaccine; MCV, Measles-Containing Vaccine; OPV, Oral Polio Vaccine; PCV, Pneumococcal Vaccine; RV, Rotavirus vaccine. (PDF) [file pgph.0003749.s009.pdf]

Table S2: Year of vaccine introduction and DHS most recent survey for each country.

	HepB	HepB-BD	Hib	IPV-D1	IPV-D2	MCV-D2	PCV	RV
**Angola (2016)**	2006	2015	2006	2017	2022	2015	2013	2014
**Bangladesh (2018)**	2005	NA	2009	2015	NA	2012	2015	NA
**Benin (2018)**	2002	2020	2005	2015	NA	NA	2011	2019
**Burkina Faso (2021)**	2006	2022	2006	2018	2021	2014	2013	2013
**Burundi (2017)**	2004	NA	2004	2015	NA	2013	2011	2013
**Cambodia (2022)**	2006	2003	2010	2015	NA	2012	2015	NA
**Cameroon (2019)**	2005	NA	2009	2015	NA	2019	2011	2014
**Chad (2015)**	2008	NA	2008	2015	2021	2022	NA	NA
**Comoros (2012)**	2003	NA	2009	2015	NA	2021	NA	NA
**Congo (2012)**	2007	NA	2009	2016	NA	2019	2012	2014
**Congo Democratic Republic (2014)**	2007	NA	2009	2015	2022	2022	2013	2019
**Cote d'Ivoire (2021)**	2003	2019	2009	2015	NA	2021	2014	2017
**Dominican Republic (2013)**	2000	2000	2002	2015	2022	2018	2013	2012
**Egypt (2014)**	2000	2015	2014	2018	2021	2000	NA	NA
**Ethiopia (2011)**	2007	NA	2007	2015	NA	2019	2011	2014
**Gabon (2021)**	2004	NA	2010	2015	NA	NA	NA	NA
**Ghana (2014)**	2002	NA	2002	2018	NA	2012	2012	2012
**Guatemala (2015)**	2005	2010	2005	2016	2020	2016	2012	2010
**Guinea (2018)**	2006	NA	2008	2015	NA	2022	NA	NA
**Haiti (2017)**	2012	NA	2012	2015	NA	2016	2018	2014
**Honduras (2012)**	2000	2008	2000	2015	2018	2018	2011	2009
**India (2021)**	2011	2008	2015	2015	NA	2011	2021	2019
**Indonesia (2017)**	2003	2000	2014	2016	NA	2005	2022	NA
**Jordan (2018)**	2000	NA	2001	2005	2006	2000	NA	2015
**Kenya (2022)**	2001	NA	2001	2015	NA	2013	2011	2014
**Kyrgyz Republic (2012)**	2001	2000	2009	2018	2022	2000	2016	2019
**Lesotho (2014)**	2003	NA	2008	2016	NA	2000	2015	2017
**Liberia (2020)**	2008	NA	2008	2017	NA	2019	2014	2016
**Madagascar (2021)**	2002	NA	2008	2015	2021	2020	2012	2014
**Malawi (2016)**	2002	NA	2002	2018	NA	2015	2011	2012
**Maldives (2017)**	2000	2000	2013	2015	NA	2007	NA	NA
**Mali (2018)**	2002	NA	2007	2016	2021	2019	2011	2015
**Mauritania (2021)**	2005	2013	2009	2015	NA	NA	2013	2014
**Myanmar (2016)**	2005	2004	2012	2015	NA	2012	2016	2020
**Namibia (2013)**	2009	2014	2009	2015	NA	2017	2014	2014
**Niger (2012)**	2008	NA	2008	2015	2022	2014	2014	2014
**Nigeria (2021)**	2004	2004	2013	2015	2021	2020	2017	2022
**Pakistan (2018)**	2002	NA	2009	2015	2021	2009	2014	2018
**Peru (2012)**	2005	2000	2005	2013	2013	2007	2009	2009
**Philippines (2022)**	2000	2006	2012	2019	NA	2010	2020	NA
**Rwanda (2020)**	2002	NA	2002	2018	2022	2014	2009	2012
**Senegal (2019)**	2004	2016	2005	2015	NA	2014	2013	2014
**Sierra Leone (2019)**	2007	NA	2007	2018	2021	2015	2011	2014
**South Africa (2016)**	2000	NA	2000	2009	2009	2000	2009	2009
**Tajikistan (2017)**	2002	2000	2008	2018	2022	2000	2022	2015
**Tanzania (2016)**	2002	NA	2009	2018	NA	2014	2013	2013
**The Gambia (2020)**	NA	NA	NA	NA	NA	NA	NA	NA
**Timor-Leste (2016)**	2007	2016	2012	2016	NA	2016	NA	2019
**Togo (2014)**	2008	NA	2008	2018	2022	2019	2014	2014
**Turkey (2019)**	2000	2003	2006	2008	2008	2000	2008	NA
**Uganda (2016)**	2002	2022	2002	2016	2022	2022	2014	2018
**Yemen (2013)**	2000	NA	2005	2015	2021	2004	2011	2012
**Zambia (2019)**	2005	NA	2004	2018	NA	2013	2013	2013
**Zimbabwe (2015)**	2000	NA	2008	2019	2022	2015	2012	2014
