# Supplementary material for: Timeliness of 24 childhood immunisations and evolution of vaccination delay: Analysis of data from 54 low- and middle-income countries
Source: PLOS Glob Public Health. 2024 Nov 26;4(11):e0003749. doi: 10.1371/journal.pgph.0003749 (PMC11593752; doi:10.1371/journal.pgph.0003749)
Supplement: S3 Table — For each vaccine, we constructed a multivariable Cox regression model using stepwise-variable selection methods: “selectCox” [31] (backwards selection) and “stepwiseCox” [32] (forward selection). The variables marked with an “x” are the ones selected by each method for each vaccine’s multivariable model. Abbreviations: BCG, Bacillus Calmette-Guérin; BD, Birth Dose; D1/2/3, Doses 1, 2 or 3; DTP, Diphtheria-Tetanus-Pertussis; HepB, Hepatitis B vaccine; Hib, Haemophilus influenzae vaccine; IPV, Inactivated Polio Vaccine; IQR, Interquartile Range; MCV, Measles-Containing Vaccine; OPV, Oral Polio Vaccine; PCV, Pneumococcal Vaccine; RV, Rotavirus vaccine. (PDF) [file pgph.0003749.s010.pdf]

Table S3: Variables selected during model selection.

	Model selection with “selectCox”						Model selection with “stepwiseCox”					
	Sex at birth	Urban/Rural	Wealth Quintile	Mother's Education	Mother's Marital Status	Husband's Occupation	Sex at birth	Urban/Rural	Wealth Quintile	Mother's Education	Mother's Marital Status	Husband's Occupation
**BCG**		x	x	x		x		x	x	x	x	x
**DTP-D1**	x	x	x	x	x	x	x	x	x	x	x	x
**DTP-D2**	x	x	x	x	x	x	x	x	x	x	x	x
**DTP-D3**	x	x	x	x	x	x	x	x	x	x	x	x
**OPV-BD**		x	x	x	x	x		x	x	x	x	x
**OPV-D1**			x	x	x	x		x	x	x	x	x
**OPV-D2**			x	x	x	x			x	x	x	x
**OPV-D3**	x	x	x	x	x	x	x	x	x	x	x	x
**IPV-D1**			x	x	x	x		x	x	x	x	x
**MCV-D1**		x	x	x		x		x	x	x	x	x
**MCV-D2**	x	x	x	x		x	x	x	x	x		x
**HepB-BD**	x	x	x	x	x	x	x	x	x	x	x	x
**HepB-D1**		x	x	x	x	x		x	x	x	x	x
**HepB-D2**		x	x	x	x	x		x	x	x	x	x
**HepB-D3**		x	x	x	x	x		x	x	x	x	x
**Hib-D1**			x	x		x			x	x		x
**Hib-D2**		x	x	x	x	x		x	x	x	x	x
**Hib-D3**		x	x	x		x		x	x	x		x
**PCV-D1**	x	x	x	x	x	x	x	x	x	x	x	x
**PCV-D2**		x	x	x	x	x		x	x	x	x	x
**PCV-D3**		x	x	x	x	x		x	x	x	x	x
**RV-D1**		x	x		x	x		x	x	x	x	x
**RV-D2**			x	x	x	x		x	x	x	x	x
**RV-D3**		x	x	x	x	x		x	x	x	x	x
