# Supplementary material for: Timeliness of 24 childhood immunisations and evolution of vaccination delay: Analysis of data from 54 low- and middle-income countries
Source: PLOS Glob Public Health. 2024 Nov 26;4(11):e0003749. doi: 10.1371/journal.pgph.0003749 (PMC11593752; doi:10.1371/journal.pgph.0003749)
Supplement: S4 Table — Total number of live children captured in all the surveys, number of children captured in each country’s survey, and number of children born in each calendar year among all countries’ surveys. For each country, year of survey in indicated. Median age at time of survey is shown in months. Abbreviations: IQR, Interquartile Range. (PDF) [file pgph.0003749.s011.pdf]

Table S4: Number of children captured in the surveys, per country and per birth cohort.

	Surveyed children, No.	Male, No.	Female, No.	Median age (IQR)
**Children included in all surveys:**	743,694	379,193	364,501	30 (14, 45)
**Children included in each country survey:**				
**Angola (2016)**	13,619	6,739	6,880	29 (14, 44)
**Bangladesh (2018)**	8,402	4,373	4,029	29 (14, 45)
**Benin (2018)**	12,651	6,383	6,268	29 (13, 45)
**Burkina Faso (2021)**	11,853	5,971	5,882	31 (15, 46)
**Burundi (2017)**	12,472	6,287	6,185	29 (14, 46)
**Cambodia (2022)**	8,025	4,085	3,940	29 (14, 45)
**Cameroon (2019)**	9,085	4,585	4,500	29 (14, 44)
**Chad (2015)**	16,901	8,509	8,392	31 (14, 46)
**Comoros (2012)**	3,022	1,519	1,503	28 (13, 44)
**Congo (2012)**	8,857	4,465	4,392	28 (13, 44)
**Congo Democratic Republic (2014)**	17,228	8,536	8,692	28 (13, 44)
**Cote d'Ivoire (2021)**	9,948	5,016	4,932	30 (14, 45)
**Dominican Republic (2013)**	4,479	2,280	2,199	30 (14, 46)
**Egypt (2014)**	15,466	8,050	7,416	28 (14, 44)
**Ethiopia (2011)**	5,414	2,774	2,640	30 (15, 45)
**Gabon (2021)**	6,111	3,145	2,966	29 (14, 45)
**Ghana (2014)**	5,595	2,905	2,690	28 (14, 44)
**Guatemala (2015)**	12,071	6,236	5,835	29 (14, 45)
**Guinea (2018)**	7,273	3,741	3,532	28 (13, 44)
**Haiti (2017)**	6,120	3,068	3,052	30 (14, 46)
**Honduras (2012)**	10,592	5,498	5,094	28 (14, 44)
**India (2021)**	224,218	115,825	108,393	31 (15, 46)
**Indonesia (2017)**	17,304	8,885	8,419	29 (15, 46)
**Jordan (2018)**	10,475	5,324	5,151	30 (14, 47)
**Kenya (2022)**	18,836	9,582	9,254	30 (14, 45)
**Kyrgyz Republic (2012)**	4,247	2,185	2,062	27 (12, 43)
**Lesotho (2014)**	2,915	1,436	1,479	26 (12, 43)
**Liberia (2020)**	5,245	2,578	2,667	30 (14, 45)
**Madagascar (2021)**	11,780	5,958	5,822	29 (14, 45)
**Malawi (2016)**	16,462	8,232	8,230	30 (15, 46)
**Maldives (2017)**	3,055	1,557	1,498	32 (16, 46)
**Mali (2018)**	9,275	4,696	4,579	29 (14, 44)
**Mauritania (2021)**	11,176	5,647	5,529	30 (14, 46)
**Myanmar (2016)**	4,597	2,402	2,195	29 (14, 45)
**Namibia (2013)**	4,818	2,377	2,441	28 (13, 44)
**Niger (2012)**	11,602	5,842	5,760	28 (13, 44)
**Nigeria (2021)**	41,358	20,991	20,367	30 (15, 45.75)
**Pakistan (2018)**	11,989	6,114	5,875	30 (15, 46)
**Peru (2012)**	9,445	4,788	4,657	30 (15, 46)
**Philippines (2022)**	8,296	4,336	3,960	32 (17, 46)
**Rwanda (2020)**	7,796	3,942	3,854	30 (15, 45)
**Senegal (2019)**	5,899	2,917	2,982	29 (14, 45)
**Sierra Leone (2019)**	9,063	4,577	4,486	28 (14, 44)
**South Africa (2016)**	3,413	1,753	1,660	31 (15, 46)
**Tajikistan (2017)**	6,019	3,036	2,983	30 (15, 45)
**Tanzania (2016)**	9,713	4,852	4,861	28 (14, 44)
**The Gambia (2020)**	7,927	4,122	3,805	28 (13, 44)
**Timor-Leste (2016)**	6,956	3,609	3,347	30 (14, 44.25)
**Togo (2014)**	6,535	3,282	3,253	28 (14, 45)
**Turkey (2019)**	2,712	1,369	1,343	32 (15, 47)
**Uganda (2016)**	14,710	7,384	7,326	30 (14, 45)
**Yemen (2013)**	15,383	7,883	7,500	28 (13, 44)
**Zambia (2019)**	9,484	4,698	4,786	29 (14, 45)

**Zimbabwe (2015)**	5,807	2,849	2,958	30 (15, 46)
**Children included in all surveys born in each calendar year:**				
**2006**	489	254	235	59 (58, 60)
**2007**	6,128	3,167	2,961	55 (52, 58)
**2008**	11,587	5,910	5,677	47 (42, 52)
**2009**	21,450	10,819	10,631	45 (34, 54)
**2010**	31,431	15,927	15,504	41 (25, 50)
**2011**	41,575	21,153	20,422	34 (19, 49)
**2012**	44,962	22,560	22,402	33 (18, 47)
**2013**	54,156	27,325	26,831	32 (14, 49)
**2014**	62,297	31,811	30,486	38 (19, 51)
**2015**	86,955	44,131	42,824	43 (25, 52)
**2016**	91,688	47,174	44,514	38 (23, 46)
**2017**	95,255	48,702	46,553	29 (18, 42)
**2018**	82,221	42,015	40,206	20 (13, 33)
**2019**	63,352	32,618	30,734	12 (5, 24)
**2020**	33,035	16,953	16,082	11 (6, 17)
**2021**	15,322	7,750	7,572	6 (2, 9)
**2022**	1,791	924	867	2 (1, 3)
