# Supplementary material for: Timeliness of 24 childhood immunisations and evolution of vaccination delay: Analysis of data from 54 low- and middle-income countries
Source: PLOS Glob Public Health. 2024 Nov 26;4(11):e0003749. doi: 10.1371/journal.pgph.0003749 (PMC11593752; doi:10.1371/journal.pgph.0003749)
Supplement: S5 Table — Proportion of countries with/without the vaccine introduced was estimated as the number of countries in which the vaccine had / had not been introduced by the time of the survey among all 54 included in the analysis. Percentage of children from countries in which each vaccine had been introduced by the time of the survey was calculated over the total number of alive children captured in the surveys (n = 743,694, S4 Table). Proportion of children with vaccination data for each vaccine was estimated over all those children from countries in which the vaccine had been introduced. Proportion of children with at least one year buffer time (time between recommended date of vaccination and survey was ≥1 year) was estimated over all of those with vaccination data. Percentage of vaccinated children was calculated over all of those with vaccination data with one year buffer time. Percentage of children with vaccination age (date) data was estimated over all those vaccinated. Abbreviations: BCG, Bacillus Calmette-Guérin; BD, Birth Dose; D1/2/3, Doses 1, 2 or 3; DTP, Diphtheria-Tetanus-Pertussis; HepB, Hepatitis B vaccine; Hib, Haemophilus influenzae vaccine; IPV, Inactivated Polio Vaccine; MCV, Measles-Containing Vaccine; OPV, Oral Polio Vaccine; PCV, Pneumococcal Vaccine; RV, Rotavirus vaccine. (PDF) [file pgph.0003749.s012.pdf]

Table S5: Number of countries and children with data available for each vaccine.

Vaccine	Countries with vaccine introduced, No. (%)	Countries with vaccine not introduced, No. (%)	Countries with vaccine introduced with vaccine data, No. (%)	Children from countries with vaccine introduced, No. (%)	Children with vaccine data, No. (%)	Children with vaccine data with 1-year buffer time, No. (%)	Vaccinated children, No. (%)	Vaccinated children with vaccination age data, No. (%)
**BCG**	54 (100)	0 (0)	54 (100)	743,694 (100)	507,599 (68.25)	356,499 (70.23)	314,584 (88.24)	205,332 (65.27)
**DTP-D1**	54 (100)	0 (0)	54 (100)	743,694 (100)	497,298 (66.87)	343,315 (69.04)	295,501 (86.07)	186,515 (63.12)
**DTP-D2**	54 (100)	0 (0)	54 (100)	743,694 (100)	496,490 (66.76)	341,298 (68.74)	279,831 (81.99)	192,523 (68.8)
**DTP-D3**	54 (100)	0 (0)	54 (100)	743,694 (100)	496,181 (66.72)	339,785 (68.48)	258,753 (76.15)	184,996 (71.5)
**HepB-BD**	20 (37)	8 (15)	14 (70)	670,394 (90.14)	216,095 (32.23)	148,182 (68.57)	91,250 (61.58)	65,955 (72.28)
**HepB-D1**	54 (100)	0 (0)	38 (70)	743,694 (100)	349,515 (47)	229,555 (65.68)	197,416 (86)	146,665 (74.29)
**HepB-D2**	54 (100)	0 (0)	38 (70)	743,694 (100)	349,382 (46.98)	227,964 (65.25)	186,960 (82.01)	140,799 (75.31)
**HepB-D3**	54 (100)	0 (0)	38 (70)	743,694 (100)	349,361 (46.98)	227,158 (65.02)	174,075 (76.63)	134,567 (77.3)
**Hib-D1**	53 (98)	1 (2)	35 (66)	728,228 (97.92)	211,668 (29.07)	138,082 (65.24)	114,923 (83.23)	76,783 (66.81)
**Hib-D2**	53 (98)	1 (2)	35 (66)	728,228 (97.92)	211,649 (29.06)	137,199 (64.82)	107,456 (78.32)	74,229 (69.08)
**Hib-D3**	53 (98)	1 (2)	35 (66)	728,228 (97.92)	211,632 (29.06)	136,334 (64.42)	98,974 (72.6)	70,875 (71.61)
**IPV-D1**	30 (56)	26 (48)	20 (67)	505,802 (68.01)	122,972 (24.31)	78,597 (63.91)	48,620 (61.86)	27,996 (57.58)
**IPV-D2**	4 (7)	24 (44)	2 (50)	468,549 (63)	9,857 (2.1)	9,857 (100)	0 (0)	NA
**MCV-D1**	54 (100)	0 (0)	54 (100)	743,694 (100)	467,728 (62.89)	314,731 (67.29)	254,041 (80.72)	160,201 (63.06)
**MCV-D2**	34 (63)	24 (44)	24 (71)	525,967 (70.72)	284,772 (54.14)	172,511 (60.58)	83,522 (48.42)	57,664 (69.04)
**OPV-BD**	54 (100)	0 (0)	43 (80)	743,694 (100)	432,403 (58.14)	309,083 (71.48)	199,490 (64.54)	135,061 (67.7)
**OPV-D1**	54 (100)	0 (0)	54 (100)	743,694 (100)	498,512 (67.03)	350,299 (70.27)	279,876 (79.9)	183,460 (65.55)
**OPV-D2**	54 (100)	0 (0)	54 (100)	743,694 (100)	496,986 (66.83)	347,337 (69.89)	261,407 (75.26)	177,259 (67.81)
**OPV-D3**	54 (100)	0 (0)	54 (100)	743,694 (100)	496,780 (66.8)	342,465 (68.94)	237,525 (69.36)	180,104 (75.83)
**PCV-D1**	34 (63)	16 (30)	32 (94)	418,724 (56.3)	206,592 (49.34)	138,471 (67.03)	102,072 (73.71)	69,413 (68)
**PCV-D2**	34 (63)	16 (30)	32 (94)	418,724 (56.3)	206,348 (49.28)	137,456 (66.61)	94,103 (68.46)	68,145 (72.42)
**PCV-D3**	34 (63)	16 (30)	32 (94)	418,724 (56.3)	197,454 (47.16)	129,455 (65.56)	85,934 (66.38)	62,129 (72.3)
**RV-D1**	30 (56)	16 (30)	31 (103)	487,113 (65.5)	315,646 (64.8)	213,709 (67.71)	124,982 (58.48)	92,532 (74.04)
**RV-D2**	30 (56)	16 (30)	31 (103)	487,113 (65.5)	315,379 (64.74)	212,283 (67.31)	113,655 (53.54)	86,179 (75.83)
**RV-D3**	30 (56)	16 (30)	9 (30)	487,113 (65.5)	175,283 (35.98)	122,679 (69.99)	39,901 (32.52)	32,336 (81.04)
