# Supplementary material for: Timeliness of 24 childhood immunisations and evolution of vaccination delay: Analysis of data from 54 low- and middle-income countries
Source: PLOS Glob Public Health. 2024 Nov 26;4(11):e0003749. doi: 10.1371/journal.pgph.0003749 (PMC11593752; doi:10.1371/journal.pgph.0003749)
Supplement: S6 Table — Vaccination delay is defined as the difference between age at vaccination and recommended age for vaccination, in weeks. For each country, year of survey in indicated. Abbreviations: BCG, Bacillus Calmette-Guérin; BD, Birth Dose; D1/2/3, Doses 1, 2 or 3; DTP, Diphtheria-Tetanus-Pertussis; HepB, Hepatitis B vaccine; Hib, Haemophilus influenzae vaccine; IPV, Inactivated Polio Vaccine; IQR, Interquartile Range; MCV, Measles-Containing Vaccine; OPV, Oral Polio Vaccine; PCV, Pneumococcal Vaccine; RV, Rotavirus vaccine. (PDF) [file pgph.0003749.s013.pdf]

Table S6: Median vaccination delay (and IQR) per country.

	BCG	DTP-D1	DTP-D2	DTP-D3	HepB-BD	HepB-D1	HepB-D2	HepB-D3	Hib-D1	Hib-D2	Hib-D3	IPV-D1
**All**	1 (0, 4)	1 (0, 4)	3 (1, 6)	4 (2, 9)	0 (0, 0)	1 (0, 4)	3 (1, 6)	4 (2, 9)	1 (0, 3)	2 (1, 5)	3 (1, 7)	4 (1, 10)
**Angola (2016)**	1 (0, 5)	1 (0, 4)	2 (1, 6)	1 (0, 6)	1 (0, 9)	1 (0, 4)	2 (1, 6)	1 (0, 6)	1 (0, 4)	2 (1, 6)	1 (0, 6)	NA
**Bangladesh (2018)**	7 (4, 9)	2 (1, 4)	3 (2, 6)	4 (2, 8)	NA	2 (1, 4)	3 (2, 6)	4 (2, 8)	2 (1, 4)	3 (2, 6)	4 (2, 8)	13 (10, 18)
**Benin (2018)**	1 (0, 2)	1 (0, 2)	2 (1, 4)	3 (1, 7)	NA	1 (0, 2)	2 (1, 4)	3 (1, 7)	1 (0, 2)	2 (1, 4)	3 (1, 7)	NA
**Burkina Faso (2021)**	1 (1, 2)	1 (1, 3)	2 (1, 4)	3 (2, 6)	NA	1 (1, 3)	2 (1, 4)	3 (2, 6)	1 (1, 3)	2 (1, 4)	3 (2, 6)	4 (2, 7)
**Burundi (2017)**	1 (1, 2)	1 (0, 2)	2 (1, 3)	3 (2, 4)	NA	1 (0, 2)	2 (1, 3)	3 (2, 4)	1 (0, 2)	2 (1, 3)	3 (2, 4)	NA
**Cambodia (2022)**	0 (0, 0)	1 (1, 1)	1 (1, 2)	2 (2, 4)	0 (0, 0)	1 (1, 1)	1 (1, 2)	2 (2, 4)	1 (1, 1)	1 (1, 2)	2 (2, 4)	2 (2, 4)
**Cameroon (2019)**	1 (0, 4)	1 (0, 2)	1 (0, 4)	2 (1, 5)	NA	1 (0, 2)	1 (0, 4)	2 (1, 5)	1 (0, 2)	1 (0, 4)	2 (1, 5)	NA
**Chad (2015)**	6 (2, 23)	5 (1, 25)	8 (2, 25)	11 (4, 28.5)	NA	NA	NA	NA	NA	NA	NA	NA
**Comoros (2012)**	1 (0, 2)	2 (0, 5)	3 (1, 8.25)	5 (2, 14)	NA	NA	NA	NA	NA	NA	NA	NA
**Congo (2012)**	2 (0, 4)	2 (0, 4)	3 (1, 6)	4 (1, 8)	NA	NA	NA	NA	NA	NA	NA	NA
**Congo Democratic Republic (2014)**	1 (0, 5)	2 (0, 5)	3 (0, 8)	4 (1, 10)	NA	NA	NA	NA	NA	NA	NA	NA
**Cote d'Ivoire (2021)**	1 (0, 3)	1 (0, 3)	2 (1, 5)	3 (1, 8)	NA	1 (0, 3)	2 (1, 5)	3 (1, 8)	1 (0, 3)	2 (1, 5)	3 (1, 8)	3 (1, 8)
**Dominican Republic (2013)**	1 (0, 2)	1 (0, 2)	2 (1, 5)	3 (1, 7)	NA	NA	NA	NA	NA	NA	NA	NA
**Egypt (2014)**	1 (0, 3)	0 (-1, 1)	1 (0, 2)	1 (0, 2)	NA	NA	NA	NA	NA	NA	NA	NA
**Ethiopia (2011)**	6 (1, 11)	1 (0, 5)	2 (1, 6)	3 (1, 7)	NA	1 (0, 5)	2 (1, 6)	3 (1, 7)	1 (0, 5)	2 (1, 6)	3 (1, 7)	3 (1, 8)
**Gabon (2021)**	1 (0, 3)	1 (0, 2)	2 (1, 4)	2 (1, 6)	NA	1 (0, 2)	2 (1, 4)	2 (1, 6)	1 (0, 2)	2 (1, 4)	2 (1, 6)	2 (1, 6)
**Ghana (2014)**	2 (0, 4)	2 (0, 3)	3 (1, 5)	4 (2, 7.25)	NA	NA	NA	NA	NA	NA	NA	NA
**Guatemala (2015)**	2 (0, 5)	1 (-1, 4)	3 (0, 6)	4 (1, 9)	NA	NA	NA	NA	NA	NA	NA	NA
**Guinea (2018)**	1 (0, 4)	1 (0, 5)	2 (1, 8)	4 (1, 11)	NA	1 (0, 5)	2 (1, 8)	4 (1, 11)	1 (0, 5)	2 (1, 8)	4 (1, 11)	13.5 (3, 25)
**Haiti (2017)**	4 (1, 10.25)	3 (1, 8)	6 (2, 13)	9 (4, 19)	NA	3 (1, 8)	6 (2, 13)	9 (4, 19)	3 (1, 8)	6 (2, 13)	9 (4, 19)	NA
**Honduras (2012)**	1 (0, 3)	1 (-1, 2)	2 (0, 4)	2 (1, 5)	NA	NA	NA	NA	NA	NA	NA	NA
**India (2021)**	0 (0, 2)	2 (1, 5)	4 (2, 7)	5 (3, 11)	0 (0, 0)	2 (1, 4)	4 (2, 7)	5 (3, 11)	NA	NA	NA	NA
**Indonesia (2017)**	5 (4, 8)	3 (0, 5)	4 (1, 8)	5 (2, 10)	1 (-1, 2)	3 (0, 5)	4 (1, 8)	5 (2, 10)	3 (0, 5)	4 (1, 8)	5 (2, 10)	NA
**Jordan (2018)**	4 (2, 5)	-3 (-4, 0)	-1 (-3, 1)	-1 (-3, 3)	NA	-3 (-4, 0)	-1 (-3, 1)	0 (-2, 4)	-3 (-4, 0)	-1 (-3, 1)	0 (-2, 4)	NA
**Kenya (2022)**	0 (0, 2)	0 (0, 1)	1 (0, 2)	1 (1, 3)	NA	0 (0, 1)	1 (0, 2)	1 (1, 3)	0 (0, 1)	1 (0, 2)	1 (1, 3)	1 (0, 3)
**Kyrgyz Republic (2012)**	1 (-1, 2)	1 (-1, 2)	4 (2, 6)	3 (1, 6)	NA	-9 (-10, -8)	1 (0, 3)	12 (9, 15)	NA	NA	NA	NA
**Lesotho (2014)**	2 (0, 5)	1 (0, 3)	3 (1, 5)	4 (2, 7)	NA	NA	NA	NA	NA	NA	NA	NA
**Liberia (2020)**	1 (0, 3)	1 (0, 3)	2 (1, 6)	4 (2, 11)	NA	1 (0, 3)	2 (1, 6)	4 (2, 11)	1 (0, 3)	2 (1, 6)	4 (2, 11)	6 (2, 18)
**Madagascar (2021)**	3 (1, 6)	1 (0, 4)	2 (1, 5)	3 (1, 6)	NA	1 (0, 4)	2 (1, 5)	3 (1, 6)	1 (0, 4)	2 (1, 5)	3 (1, 6)	3 (1, 7)

**Malawi (2016)**	1 (0, 4)	2 (1, 4)	3 (1, 5)	4 (2, 8)	NA	2 (1, 4)	3 (1, 5)	4 (2, 8)	2 (1, 4)	3 (1, 5)	4 (2, 8)	NA
**Maldives (2017)**	0 (0, 0)	0 (0, 1)	1 (1, 2)	1 (0, 3)	0 (0, 0)	0 (0, 1)	1 (1, 2)	1 (0, 3)	0 (0, 1)	1 (1, 2)	1 (0, 3)	1 (0, 3)
**Mali (2018)**	3 (1, 7)	2 (1, 6)	4 (2, 9)	6 (3, 13)	NA	2 (1, 6)	4 (2, 9)	6 (3, 13)	2 (1, 6)	4 (2, 9)	6 (3, 13)	5 (1, 12)
**Mauritania (2021)**	1 (0, 6)	1 (0, 4)	3 (1, 8)	7 (3, 16)	0 (0, 2)	1 (0, 4)	3 (1, 8)	7 (3, 16)	1 (0, 4)	3 (1, 8)	7 (3, 16)	6 (2, 14)
**Myanmar (2016)**	11 (7, 12)	2 (-1, 6)	3 (-1, 7)	3 (-1, 7)	2 (0, 11)	2 (-1, 6)	3 (-1, 7)	3 (-1, 7)	NA	NA	NA	NA
**Namibia (2013)**	1 (-1, 2)	1 (-1, 2)	1 (0, 3)	2 (0, 4)	NA	NA	NA	NA	NA	NA	NA	NA
**Niger (2012)**	3 (1, 9)	3 (0, 7)	5 (2, 11)	7 (3, 16)	NA	NA	NA	NA	NA	NA	NA	NA
**Nigeria (2021)**	2 (1, 5)	1 (0, 5)	2 (0, 6)	3 (1, 9)	1 (0, 3)	1 (0, 5)	2 (0, 6)	3 (1, 9)	1 (0, 5)	2 (0, 6)	3 (1, 9)	12 (9, 20)
**Pakistan (2018)**	2 (1, 4)	2 (1, 4)	3 (2, 7)	5 (2, 10)	NA	2 (1, 4)	3 (2, 7)	5 (2, 10)	2 (1, 4)	3 (2, 7)	5 (2, 10)	5 (2, 10)
**Peru (2012)**	1 (0, 3)	1 (-1, 2)	2 (1, 5)	2 (0, 6)	NA	NA	NA	NA	NA	NA	NA	NA
**Philippines (2022)**	0 (0, 4)	2 (1, 4)	4 (2, 7)	5 (3, 11)	0 (0, 0)	2 (1, 4)	4 (2, 7)	5 (3, 11)	2 (1, 4)	4 (2, 7)	5 (3, 11)	6 (3, 12)
**Rwanda (2020)**	1 (0, 2)	1 (0, 2)	1 (0, 2)	2 (1, 3)	NA	1 (0, 2)	1 (0, 2)	2 (1, 3)	1 (0, 2)	1 (0, 2)	2 (1, 3)	1 (1, 3)
**Senegal (2019)**	2 (1, 4)	2 (1, 4)	3 (1, 7)	5 (2, 11)	0 (0, 2)	2 (1, 4)	3 (1, 7)	5 (2, 11)	2 (1, 4)	3 (1, 7)	5 (2, 11)	NA
**Sierra Leone (2019)**	1 (0, 2)	1 (0, 3)	3 (1, 6)	4 (2, 10)	NA	1 (0, 3)	3 (1, 6)	4 (2, 10)	1 (0, 3)	3 (1, 6)	4 (2, 10)	5 (2, 12)
**South Africa (2016)**	0 (0, 0)	0 (0, 1)	1 (0, 3)	1 (0, 4)	NA	0 (0, 1)	1 (0, 2)	1 (0, 3)	0 (0, 1)	1 (0, 3)	1 (0, 4)	NA
**Tajikistan (2017)**	0 (0, 2)	NA	NA	NA	0 (0, 0)	NA	NA	NA	NA	NA	NA	NA
**Tanzania (2016)**	2 (0, 6)	1 (0, 3)	2 (1, 5)	4 (2, 7)	NA	1 (0, 3)	2 (1, 5)	4 (2, 7)	1 (0, 3)	2 (1, 5)	4 (2, 7)	NA
**The Gambia (2020)**	2 (1, 4)	2 (0, 3)	3 (1, 5)	4 (2, 8)	2 (1, 4)	2 (0, 3)	3 (1, 5)	4 (2, 8)	2 (0, 3)	3 (1, 5)	4 (2, 8)	4 (2, 9)
**Timor-Leste (2016)**	2 (0, 4)	1 (0, 3)	2 (1, 5)	3 (1, 8)	NA	1 (0, 3)	2 (1, 5)	3 (1, 8)	1 (0, 3)	2 (1, 5)	3 (1, 8)	NA
**Togo (2014)**	2 (1, 4)	2 (0, 4)	3 (1, 6)	4 (2, 8)	NA	NA	NA	NA	NA	NA	NA	NA
**Turkey (2019)**	0 (0, 1)	0 (0, 1)	1 (1, 2)	1 (0, 2)	NA	-4 (-4, -4)	-21 (-22, -21)	NA	NA	NA	NA	NA
**Uganda (2016)**	1 (0, 4.5)	1 (0, 4)	3 (1, 6)	4 (2, 10)	NA	1 (0, 4)	3 (1, 6)	4 (2, 10)	1 (0, 4)	3 (1, 6)	4 (2, 10)	29.5 (14, 39)
**Yemen (2013)**	7 (3, 12)	3 (0, 7)	4 (2, 10)	6 (3, 15)	NA	NA	NA	NA	NA	NA	NA	NA
**Zambia (2019)**	1 (0, 5)	1 (0, 3)	3 (1, 6)	4 (2, 9)	NA	1 (0, 3)	3 (1, 6)	4 (2, 9)	1 (0, 3)	3 (1, 6)	4 (2, 9)	NA
**Zimbabwe (2015)**	0 (0, 1)	0 (0, 1)	1 (0, 3)	2 (1, 5)	NA	0 (0, 1)	1 (0, 3)	2 (1, 5)	0 (0, 1)	1 (0, 3)	2 (1, 5)	NA

	MCV-D1	MCV-D2	OPV-BD	OPV-D1	OPV-D2	OPV-D3	PCV-D1	PCV-D2	PCV-D3	RV-D1	RV-D2	RV-D3
**All**	2 (0, 6)	4 (1, 10)	0 (0, 2)	1 (0, 4)	3 (1, 6)	4 (2, 9)	1 (0, 4)	2 (1, 5)	3 (1, 8)	1 (0, 4)	3 (1, 6)	5 (2, 10)
**Angola (2016)**	1 (0, 6)	-21 (-25, 1)	1 (0, 4)	1 (0, 4)	2 (1, 6)	2 (0, 6)	1 (0, 6.25)	2 (1, 9)	2 (0, 8)	1 (0, 4)	2 (1, 7)	NA
**Bangladesh (2018)**	0.5 (-3.75, 2.25)	4 (2, 6)	NA	2 (1, 4)	3 (2, 6)	4 (2, 8)	2 (1, 4)	3 (2, 6)	8 (6, 13)	NA	NA	NA
**Benin (2018)**	2 (1, 5)	NA	1 (0, 2)	1 (0, 2)	2 (1, 4)	3 (1, 7)	1 (0, 2)	2 (1, 4)	3 (1, 7)	NA	NA	NA
**Burkina Faso (2021)**	2 (0, 5)	3 (1, 6)	1 (0, 2)	1 (1, 3)	2 (1, 4)	3 (2, 6)	1 (1, 3)	-2 (-3, 0)	-20 (-21, -17)	2 (1, 3)	2 (1, 5)	4 (2, 6)
**Burundi (2017)**	1 (0, 3)	3 (2, 6)	1 (1, 2)	1 (0, 2)	2 (1, 3)	3 (2, 4)	1 (0, 2)	2 (1, 3)	3 (2, 4)	1 (0, 2)	2 (1, 3)	NA
**Cambodia (2022)**	1 (0, 2)	2 (-1, 4)	NA	1 (1, 1)	1 (1, 2)	2 (2, 4)	1 (1, 1)	1 (1, 3)	2 (2, 5)	NA	NA	NA
**Cameroon (2019)**	1 (0, 4)	NA	1 (0, 3)	1 (0, 2)	1 (0, 3)	2 (1, 5)	1 (0, 2)	1 (0, 4)	2 (1, 5)	1 (0, 3)	1 (0, 4)	NA
**Chad (2015)**	7 (0, 52.25)	NA	5 (1, 18)	5 (0, 23)	7 (2, 24)	11 (4, 30)	NA	NA	NA	NA	NA	NA
**Comoros (2012)**	2 (-1, 11)	NA	1 (0, 3)	2 (0, 4)	3 (1, 8)	5 (2, 13)	NA	NA	NA	NA	NA	NA
**Congo (2012)**	5 (3, 9)	NA	2 (0, 4)	2 (0, 4)	3 (1, 6)	4 (2, 8)	NA	NA	NA	NA	NA	NA
**Congo Democratic Republic (2014)**	2 (0, 8)	NA	1 (0, 4)	2 (0, 5)	3 (0, 8)	4 (1, 10)	NA	NA	NA	NA	NA	NA
**Cote d'Ivoire (2021)**	2 (0, 6)	NA	1 (0, 2)	1 (0, 3)	2 (1, 5)	3 (1, 8)	1 (0, 3)	2 (1, 5)	3 (1, 8)	1 (0, 4)	2 (1, 6)	NA
**Dominican Republic (2013)**	3 (1, 6.5)	NA	NA	NA	NA	-48 (-50, -44)	NA	NA	NA	45 (43, 49)	2 (1, 5.75)	NA
**Egypt (2014)**	0 (-1, 2)	2 (1, 4)	1 (0, 2)	0 (-1, 1)	1 (0, 2)	1 (0, 2)	NA	NA	NA	NA	NA	NA
**Ethiopia (2011)**	2 (0, 6)	3 (-6, 15)	1 (0, 2)	1 (0, 5)	2 (1, 6)	3 (1, 8)	1 (0, 4)	2 (1, 6)	3 (1, 7)	1 (0, 5)	2 (1, 6)	NA
**Gabon (2021)**	2 (1, 5)	NA	1 (0, 2)	1 (0, 2)	2 (1, 4)	2 (1, 6)	NA	NA	NA	NA	NA	NA
**Ghana (2014)**	3 (1, 6)	5 (1, 15)	1 (0, 2)	2 (0, 3)	3 (1, 5)	4 (2, 7)	2 (0, 4)	3 (1, 6)	4 (2, 7.75)	2 (0, 4)	3 (1, 6)	NA
**Guatemala (2015)**	2 (-2, 5)	NA	NA	NA	NA	4 (1, 9)	2 (0, 8)	4 (1, 10)	NA	1 (0, 4)	3 (1, 6)	NA
**Guinea (2018)**	1 (-5.5, 6)	NA	1 (0, 4)	1 (0, 5)	2 (0, 7)	3 (1, 10)	NA	NA	NA	NA	NA	NA
**Haiti (2017)**	4 (1, 13)	NA	NA	9 (7, 13)	5 (2, 11)	8 (4, 18)	NA	NA	NA	3 (1, 8)	6 (2, 13)	NA
**Honduras (2012)**	2 (0, 3)	NA	NA	NA	NA	2 (1, 5)	21 (7, 45)	11 (3, 20)	12 (4, 21)	1 (-1, 2)	2 (0, 3)	NA
**India (2021)**	3 (1, 7)	6 (2, 12)	0 (0, 0)	2 (1, 4)	4 (2, 7)	5 (3, 11)	NA	NA	NA	2 (1, 5)	4 (2, 8)	5 (3, 11)
**Indonesia (2017)**	3 (0, 6)	NA	NA	8 (5, 11)	8 (5, 13)	10 (7, 16)	NA	NA	NA	NA	NA	NA
**Jordan (2018)**	15 (14, 18)	3 (1, 7)	NA	-2 (-3, 1)	0 (-2, 2)	2 (0, 5)	NA	NA	NA	-3 (-4, 0)	-1 (-3, 1)	0 (-2, 4)
**Kenya (2022)**	2 (0, 4)	2 (1, 5)	0 (0, 1)	0 (0, 1)	1 (0, 2)	1 (1, 3)	0 (0, 1)	1 (0, 2)	1 (1, 3)	0 (0, 1)	1 (0, 2)	NA
**Kyrgyz Republic (2012)**	2 (0, 5)	NA	NA	1 (0, 4)	5 (3, 9)	4 (2, 10)	NA	NA	NA	NA	NA	NA
**Lesotho (2014)**	4 (1, 9)	NA	1 (0, 2)	2 (0, 3)	3 (1, 5)	4 (2, 8)	NA	NA	NA	NA	NA	NA
**Liberia (2020)**	2 (0, 5)	NA	0 (0, 1)	1 (0, 3)	2 (1, 6.75)	4 (2, 10)	1 (0, 3)	2.5 (1, 6)	4 (2, 10)	1 (0, 3)	3 (1, 6)	NA
**Madagascar (2021)**	1 (-1, 5)	NA	2 (1, 5)	1 (0, 4)	2 (1, 5)	3 (1, 6)	1 (0, 4)	2 (1, 5)	3 (1, 6)	1 (0, 4)	2 (1, 5)	NA
**Malawi (2016)**	3 (1, 7)	8 (-6, 21)	1 (0, 2)	2 (1, 4)	3 (1, 7)	5 (2, 11)	2 (1, 4)	3 (1, 6)	4 (2, 9)	2 (1, 4)	3 (1, 6)	NA

**Maldives (2017)**	2 (0, 3)	2 (2, 4)	NA	0 (0, 2)	1 (1, 3)	1 (0, 2)	NA	NA	NA	NA	NA	NA
**Mali (2018)**	2 (-1, 6)	NA	2 (1, 5)	2 (1, 6)	4 (2, 9)	6 (3, 13)	2 (1, 6)	4 (2, 9)	7 (3, 13)	2 (1, 7)	5 (2, 10)	7 (3, 15)
**Mauritania (2021)**	2 (-1.25, 8)	NA	0 (0, 3)	1 (0, 4)	3 (1, 7)	6 (2, 15)	1 (0, 4)	4 (1, 8)	7 (3, 16)	1 (0, 5)	4 (1, 8)	NA
**Myanmar (2016)**	3 (2, 7)	4 (0, 8)	NA	2 (-1, 6)	3 (-1, 7)	3 (-1, 7)	NA	NA	NA	NA	NA	NA
**Namibia (2013)**	1 (0, 3)	NA	1 (-1, 2)	1 (-1, 2)	1 (0, 3)	2 (0, 4)	NA	NA	NA	NA	NA	NA
**Niger (2012)**	3 (0, 10)	NA	2 (1, 6)	3 (0, 7)	5 (2, 11)	7 (3, 16)	NA	NA	NA	NA	NA	NA
**Nigeria (2021)**	1 (0, 6)	-11 (-23, 4.75)	1 (1, 3)	1 (0, 4)	2 (0, 6)	3 (1, 9)	1 (0, 6)	2 (1, 8)	3 (1, 11)	NA	NA	NA
**Pakistan (2018)**	2 (0, 6)	3 (1, 6)	2 (1, 3)	2 (1, 4)	3 (2, 7)	5 (2, 10)	2 (1, 4)	3 (2, 7)	5 (2, 10)	NA	NA	NA
**Peru (2012)**	3 (1, 8)	NA	NA	-16 (-18, -15)	-58 (-59, -55)	NA	NA	NA	NA	1 (0, 3)	2 (0, 5)	NA
**Philippines (2022)**	3 (1, 6)	2 (1, 6)	NA	2 (1, 5)	4 (2, 8)	6 (3, 11)	3 (1, 7)	4 (2, 11)	8 (4, 15)	NA	NA	NA
**Rwanda (2020)**	1 (0, 2)	2 (1, 4)	1 (0, 1)	1 (0, 2)	1 (0, 2)	1 (1, 3)	1 (0, 2)	1 (0, 2)	2 (1, 3)	1 (0, 2)	1 (1, 2)	NA
**Senegal (2019)**	2 (0, 7)	3 (1, 8)	1 (0, 2)	2 (1, 4)	3 (1, 7)	5 (2, 11)	2 (1, 4)	3 (1, 7)	5 (2, 11)	2 (1, 4)	3 (1, 7)	NA
**Sierra Leone (2019)**	2 (0, 8)	1 (-14, 6)	1 (0, 2)	1 (0, 3)	2 (1, 6)	4 (2, 10)	1 (0, 3)	3 (1, 6)	4 (2, 10)	1 (0, 3)	3 (1, 6)	NA
**South Africa (2016)**	-3 (-14, 9)	27 (25, 28)	0 (0, 0)	0 (0, 1)	NA	NA	0 (0, 1)	1 (0, 3)	1 (0, 3)	0 (0, 1)	1 (0, 3)	NA
**Tajikistan (2017)**	2 (0, 4)	NA	0 (0, 0)	NA	NA	NA	NA	NA	NA	NA	NA	NA
**Tanzania (2016)**	3 (1, 6)	4 (1, 9)	NA	1 (0, 3)	2 (1, 5)	4 (2, 8)	1 (0, 3)	2 (1, 5)	4 (2, 8)	1 (0, 3)	2 (1, 5)	NA
**The Gambia (2020)**	3 (1, 5)	5 (2, 10)	2 (1, 4)	2 (0, 3)	3 (1, 5)	4 (2, 8)	2 (0, 3)	3 (1, 5)	4 (2, 8)	2 (0, 3)	3 (1, 5)	NA
**Timor-Leste (2016)**	1 (0, 4)	NA	1 (0, 4)	1 (0, 3)	2 (1, 5)	3 (1, 8)	NA	NA	NA	NA	NA	NA
**Togo (2014)**	3 (1, 7)	NA	2 (1, 4)	2 (0, 4)	3 (1, 6)	4 (2, 8)	NA	NA	NA	NA	NA	NA
**Turkey (2019)**	1 (0, 2)	NA	NA	-17 (-17, -15)	-59 (-60, -58)	NA	0 (0, 1)	1 (1, 2)	-25 (-26, -24)	NA	NA	NA
**Uganda (2016)**	3 (1, 7)	NA	1 (0, 2)	1 (0, 4)	3 (1, 7)	4 (2, 10)	2 (1, 6)	4 (1, 10)	6 (2, 15)	5 (2, 10)	6 (2.5, 11)	NA
**Yemen (2013)**	3 (0, 16)	NA	4 (2, 7)	3 (0, 7)	4 (2, 10)	6 (3, 15)	NA	5 (2, 11)	7 (3, 16)	NA	NA	NA
**Zambia (2019)**	2 (0, 5)	3 (1, 7)	NA	1 (0, 4)	3 (1, 6)	4 (2, 9)	1 (0, 3)	3 (1, 6)	4 (2, 9)	1 (0, 4)	3 (1, 7)	NA
**Zimbabwe (2015)**	2 (0, 5)	NA	NA	0 (0, 1)	1 (0, 3)	2 (1, 6)	0 (0, 1)	1 (0, 3)	2 (1, 5)	0 (0, 2)	1 (0, 4)	NA
